# The shaping of immunological responses through natural selection after the Roma Diaspora

**DOI:** 10.1038/s41598-020-73182-1

**Published:** 2020-09-30

**Authors:** Begoña Dobon, Rob ter Horst, Hafid Laayouni, Mayukh Mondal, Erica Bianco, David Comas, Mihai Ioana, Elena Bosch, Jaume Bertranpetit, Mihai G. Netea

**Affiliations:** 1 Institut de Biologia Evolutiva (UPF-CSIC), Doctor Aiguader 88 (PRBB), Universitat Pompeu Fabra, 08003 Barcelona, Catalonia Spain; 2 Department of Internal Medicine and Radboud Center for Infectious Diseases, Radboud University Medical Center, 6525 GA Nijmegen, The Netherlands; 3 Bioinformatics Studies, ESCI-UPF, Pg. Pujades 1, 08003 Barcelona, Catalonia Spain; 4 Institute of Genomics, University of Tartu, Tartu, Estonia; 5 Department of Human Genetics, University of Medicine and Pharmacy Craiova, Craiova, Romania; 6 Centro de Investigación Biomédica en Red de Salud Mental (CIBERSAM), 43206 Reus, Spain; 7 Department for Genomics & Immunoregulation, Life and Medical Sciences Institute (LIMES), University of Bonn, 53115 Bonn, Germany; 8 Present Address: Department of Anthropology, University of Zurich, Zurich, Switzerland; 9 Present Address: CeMM Research Center for Molecular Medicine of the Austrian Academy of Sciences, Vienna, Austria

**Keywords:** Evolution, Cytokines, Infectious diseases

## Abstract

The Roma people are the largest transnational ethnic minority in Europe and can be considered the last human migration of South Asian origin into the continent. They left Northwest India approximately 1,000 years ago, reaching the Balkan Peninsula around the twelfth century and Romania in the fourteenth century. Here, we analyze whole-genome sequencing data of 40 Roma and 40 non-Roma individuals from Romania. We performed a genome-wide scan of selection comparing Roma, their local host population, and a Northwestern Indian population, to identify the selective pressures faced by the Roma mainly after they settled in Europe. We identify under recent selection several pathways implicated in immune responses, among them cellular metabolism pathways known to be rewired after immune stimulation. We validated the interaction between PIK3-mTOR-HIF-1α and cytokine response influenced by bacterial and fungal infections. Our results point to a significant role of these pathways for host defense against the most prevalent pathogens in Europe during the last millennium.

## Introduction

The Roma people, also called Romani/Rroma/Gypsies, represent the largest ethnic minority in Europe. Due to the nomadic lifestyle of some of the groups and the social exclusion that the Roma have suffered, their real census on the European continent is unclear, but estimates vary between 10 and 12 million. For reasons that are still unknown, the Roma left the Indian subcontinent around 1,000–1,500 years ago^1–3^. They traveled through Persia and Armenia, reaching the Balkan peninsula between the eleventh and twelfth centuries, where some groups settled, while other groups travelled into North, Central and Western Europe^1,4,5^. The first record of the presence of the Roma in Romanian territory dates back to 1385^6^, where they now comprise 3.3% of the population according to the last census^7^, although some authors estimate higher percentages, up to 8.6%^8^. The study of the history of the Roma, which lacks written records, has relied on anthropological, linguistic, and later, genetic studies. Roma groups follow social rules and structure similar to the Indian castes, where some groups are defined by their profession, and marriage outside the specific Roma clan is discouraged^1^. The putative Indian origin of the Roma was suggested by comparative linguistics studies that linked the Romani language to Northwestern and Central Indian languages^9,10^. Among genetic studies, uniparental markers (mitochondrial DNA and Y-chromosome haplogroups) gave further support to anthropological and linguistic studies suggesting the Indian origin of the Roma. Mitochondrial haplogroups M5a1, M18, M25 and M35b, which have a South Asian origin, are commonly found in Roma populations but not in other European populations^11–13^. Genome-wide data studies, comparing different Roma groups, further narrowed the putative population of origin of the proto-Roma to inhabit the Northwestern region of the Indian subcontinent^2,12,14–16^.

While the main topics investigated in genetic studies about the Roma concerned: i) the place of origin within the Indian subcontinent; ii) the gene flow between the Roma and the populations in their host countries, with a sex-biased component; iii) the similarities between Roma groups from different countries; and iv) the high frequency of some Mendelian diseases that constitutes a clear Roma disease heritage (see Kalaydjieva et al*.* for a review^17^); one aspect that has received less attention is which selective pressures encountered the Roma population upon migration in Europe. The Roma diaspora can be considered the last human migration of South Asian origin into Europe^4^. Thus, the evolutionary similarities driven by adaptive selection between the Roma and their local European hosts (Romanians) can help us identify very strong and recent selective pressures prevalent in the European continent.

A study focusing on genetic variation in immune genes identified important genes and pathways under selection in these two populations, leading to the identification of novel receptors for *Yersinia pestis*, the agent of plague^18^. However, a genome-wide approach is missing. In the present study, we assessed whole-genome sequences of both Roma individuals and individuals from the local European population of Romanians, to identify features of the genetic history of the Roma that point to the selective pressures they faced after settling in Europe. We identify several candidate immune and metabolic genes and pathways under recent positive selection in both Roma and non-Roma Romanians, arguing for a significant role of these pathways for host defense against infections prevalent on the continent during the last millennium of common habitation of these two populations.

## Results

### Ancestry of Roma people from Romania

**Figure 1:**
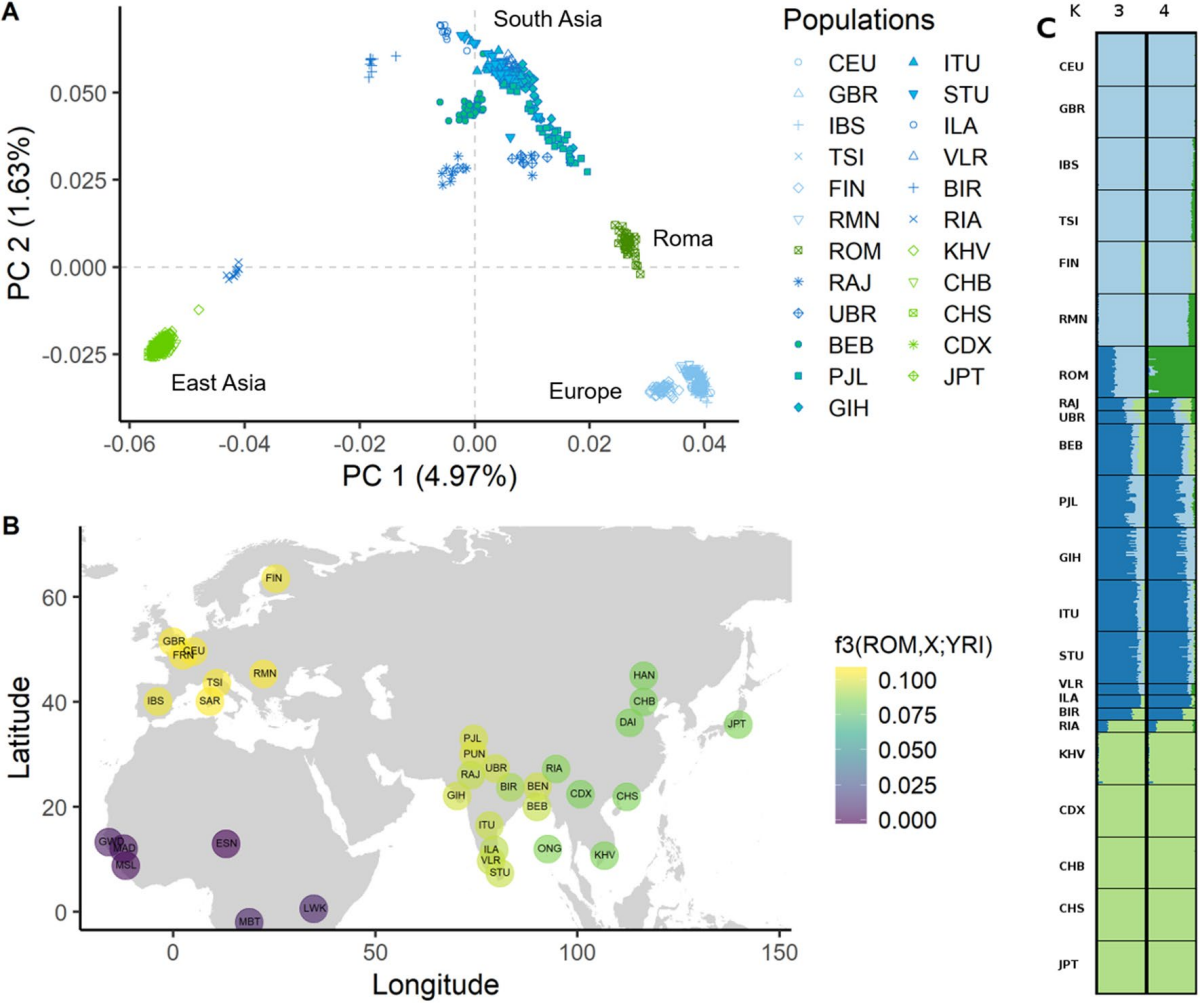
Ancestry of Roma people from Romania. (**A**) Principal component analysis of Roma (ROM) from Romania in the context of other worldwide populations. The graph shows the two principal components and the variance explained by them. Roma fall in a cline between Indian and European populations. (**B**) Proportion of shared genetic drift between Roma and extant worldwide populations measured using outgroup f3 statistic in the form f3(Roma; X, YRI). (**C**) Clustering analysis showing K = 3 and K = 4. Roma show their own component in K = 4 (best supported model). Roma share more drift with Europeans than with South East Asian populations. See Supplementary Notes for the description of the population abbreviations; some coordinates were slightly displaced to avoid overlap.

To explore the genetic relationship between the Roma (ROM) and other worldwide populations we performed a principal component analysis (PCA) after quality control of the samples (Fig. 1A, Supplementary Fig. 7). PC1 separates European and Indian populations from East Asian populations, whereas PC2 differentiates European populations (including Romanians, RMN) from Indian and Roma populations. Roma fall in a cline between European/Romanians and Indian populations, with the closest Indian populations being those geographically located in Northwest India: Rajput (RAJ), Uttar Pradesh Upper Caste Brahmins (UBR), and Punjabi (PJL). When only Roma and Romanians populations are analyzed, PC1 separates Roma from Romanians, with the later forming a tight cluster (Supplementary Fig. 6).

Although Roma groups have kept their cultural identity and status apart from the host population, we observed several individuals, both Roma and Romanian, spread between the two clusters, suggesting recent bidirectional gene flow between these populations. The proportion and direction of the admixture has been heterogeneous in the different Roma groups: the Roma carry between 65–80% of West Eurasian ancestry, with Eastern groups usually having less admixture^2,14–16^. However, previous studies suggest that the majority of the gene flow has been from European host populations to the Roma groups and higher from non-Roma males than from non-Roma females^11,16^. In an admixture analysis (Fig. 1C, Supplementary Fig. 8A), Romanian Roma appear as an admixed population with 37% South Asian and 63% European component (K = 3). It is at K = 4 when Roma show their own genetic component (best supported model, Supplementary Fig. 8B). This component can also be seen in small proportions in some European populations: Romanian, Tuscans (TSI) and Iberia (IBS), areas with a known presence of Roma; and it also appears in some Indian populations (RAJ, UBR, PJL, VLR and RIA). Subsequently, we estimated the allele sharing between Roma and other worldwide populations using the outgroup f3-statistics. Roma share more genetic drift with Central or Eastern European populations than with South Asians (Fig. 1B, Supplementary Fig. 9). It has been suggested that the West Eurasian ancestry present within India, and mainly in the North, increases the genetic similarity of the Roma with other European populations^3,14^. This, together with the influx of European migrants into a population with small effective size and the high genetic drift of the Roma subgroups, make the Roma more genetically similar to European than to South Asian populations^16^.

Global estimations of West Eurasian ancestry estimated by RFMix and ADMIXTURE (K = 3) are significantly correlated (Spearman's ρ = 0.6952, p-value = 6.436e-07). RFMix estimates an average of West Eurasian ancestry of 75.73 ± 1.81% (mean ± sd) in the Roma, whereas ADMIXTURE estimates an average of 63.36 ± 2.5% (mean ± sd). While RFMix estimates global ancestry proportions in complex admixed scenarios with higher accuracy than ADMIXTURE^19^, neither of them are formal tests of admixture and the proportions observed by them can be generated by more than one demographic process^20,21^.

To formally test for European admixture in the Romanian Roma we applied the D-statistic test in the form of D(European, African(YRI), ROM, South Asian). We did not apply the f3-statistic as a formal test of admixture, as its power to detect admixture is widely reduced when the target population has suffered strong population-specific drift after the admixture event, as is the case with the Roma^20^. Romanian Roma show significant West Eurasian admixture with all European populations tested (Supplementary Table 5). Applying the f4 ratio estimation, we estimated this admixture to be between 48–54% (Supplementary Table 6). Roma groups from the Balkan Peninsula and Central Europe tend to have lower West Eurasian ancestry than other Roma groups^16,22^.

As previously observed, uniparental markers show that Romanian Roma have less mtDNA genetic diversity than their host population (Supplementary Table 4)^11^. The main haplogroup in Romanian Roma is M5a1b (35%) identified to be of Indian origin^23^. Haplogroup M is rarely found in Europe but commonly found in Roma populations^24^. The second most common haplogroup in the Roma is H, of European origin and the main haplogroup present in non-Roma Romanians, which mostly present West Eurasian lineages: H (30%), U (22.5%), T (17.5%), K (7.5%) and J (7.5%) (Supplementary Table 4)^25^.

### Signals of bottlenecks and endogamy in Roma people

**Figure 2:**
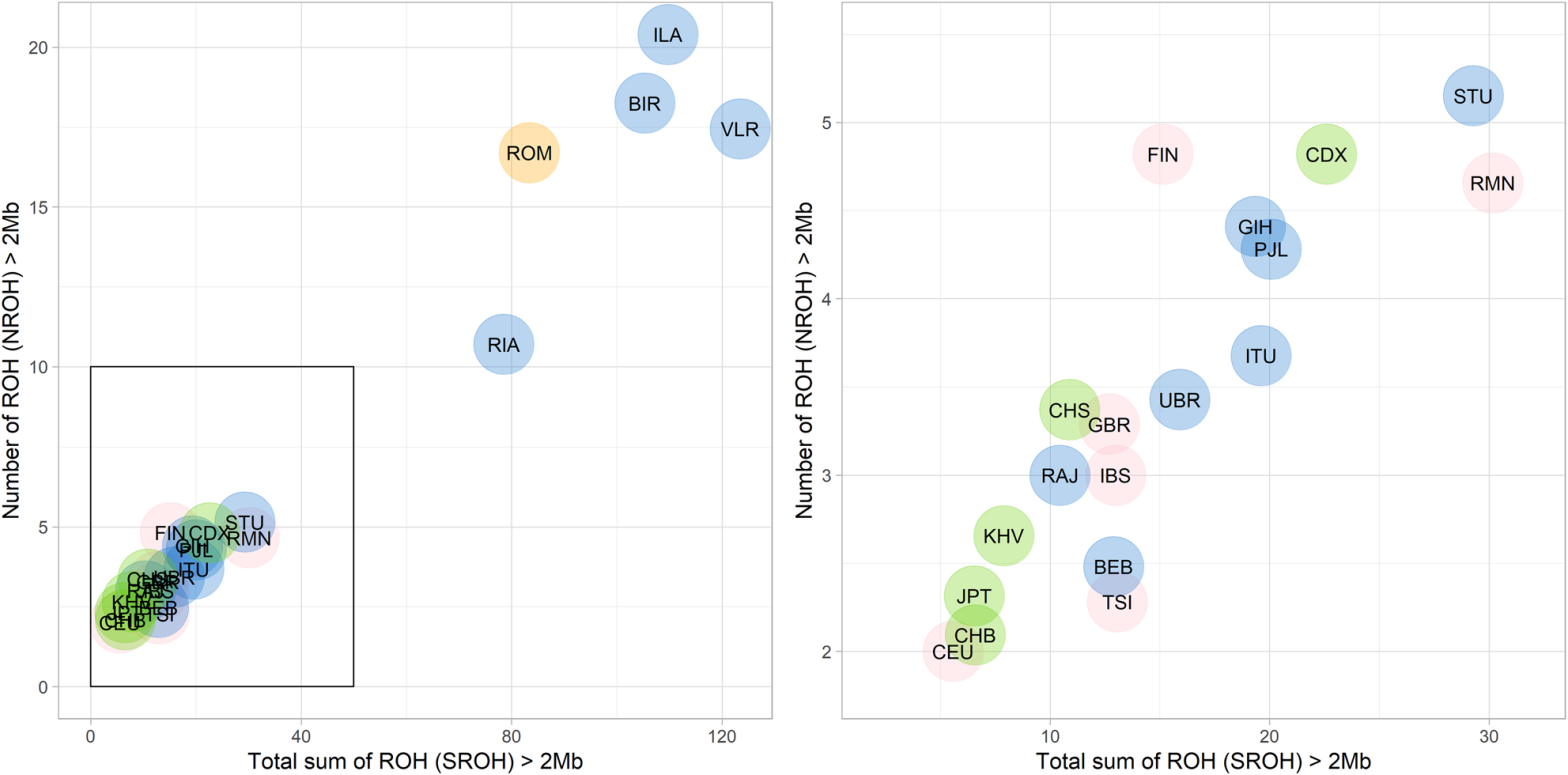
Runs of homozygosity in worldwide populations. Total number of runs of homozygosity (NROH) versus the sum of the total length of ROH in Mb (SROH) for ROH > 2 Mb in worldwide populations. Each dot represents population means. Except for Roma who are colored in orange, dots color denotes their geography: Europe (pink), South Asia (blue) and East Asia (green). Right panel is an inset of the main plot to better show the separation between populations. Roma show a similar profile to Indian tribal populations (RIA, ILA and BIR) and to populations with high endogamy, such as VLR. See Supplementary Notes for the description of the population abbreviations. See Supplementary Fig. 10d for a comparison generated by down sampling all populations to the lowest sample size available.

By comparing the number and length of runs of homozygosity (ROH) we can infer the demographic history of a population^26^. Roma show a unique profile with respect to other European populations (Fig. 2). Roma suffered a strong bottleneck after the departure from India and kept a small effective population size^2^. They have more ROHs of any given length than any other European population with higher effective population sizes, showing similar values to tribal Indian populations (Supplementary Fig. 10a-b). The practice of consanguineous marriages in the Roma^1,27^ is reflected in the high number of very long ROHs that are not equally distributed among the population, as the offspring of consanguineous unions will each have a small number of very long ROHs (Supplementary Fig. 10c). It is interesting to highlight that the genetic effects of the strong bottleneck and close endogamy are seen only in Roma and tribal Indian populations such as Riang (RIA), Birhor (BIR), and Irula (ILA), but not in the caste populations that make most of the India main gene pool (Supplementary Fig. 10c). Among the caste populations, only Vellalar (VLR) show a striking signal of inbreeding; they belong to a Dravidian caste that have a preferential cross-cousin and maternal uncle-niece marriage^28,29^ which explains the increase of very long ROHs (Supplementary Fig. 10), as seen in other South Indian Dravidian populations^30^.

Long identity-by-descent (IBD) segments can be used to infer variation in effective population size (Ne) in recent times^31^. Romanian Roma show a steadily decrease in population size, reaching a minimum around 1050 years ago (42 generations, Supplementary Fig. 11), which fits with the proposed time of the Diaspora from India^2^. There is an increase in their population size after generation 25 (1394 CE), within the range of the admixture event between the West Eurasian-like and South Asian-like sources (1270–1580) detected by Font-Porterias et al*.*^16^ and fitting with the first recorded date of the presence of Roma in Romania^6^.

### Genome distribution of the selection signals

We detected signals of recent positive selection by pairwise computing the cross-population extended haplotype homozygosity (XP-EHH) test^32^ as implemented in selscan^33^. XP-EHH detects selective sweeps in which the selected allele increased rapidly to a high frequency in one population, nearly or reaching fixation, but remains polymorphic in the other^32^, detecting strong and recent population-specific selective sweeps while comparing populations. Even though the calculation of XP-EHH is not greatly affected by complex demographic scenarios^32,34^, we performed multiple pair-wise population comparisons: Roma vs. non-Roma Romanian, Roma vs. Northwest India, and non-Roma Romanian vs. Northwest India to control for the distinct demographic histories of each population and reduce the number of false positives. This, combined with the detection of genomic regions with an excess of local European ancestry in the Roma, identified genomic regions under recent and strong positive selection shared by both Roma and Romanians and not present in Northwest India, indicative of the common selective pressures local Romanians and Roma people faced in Europe.

**Figure 3:**
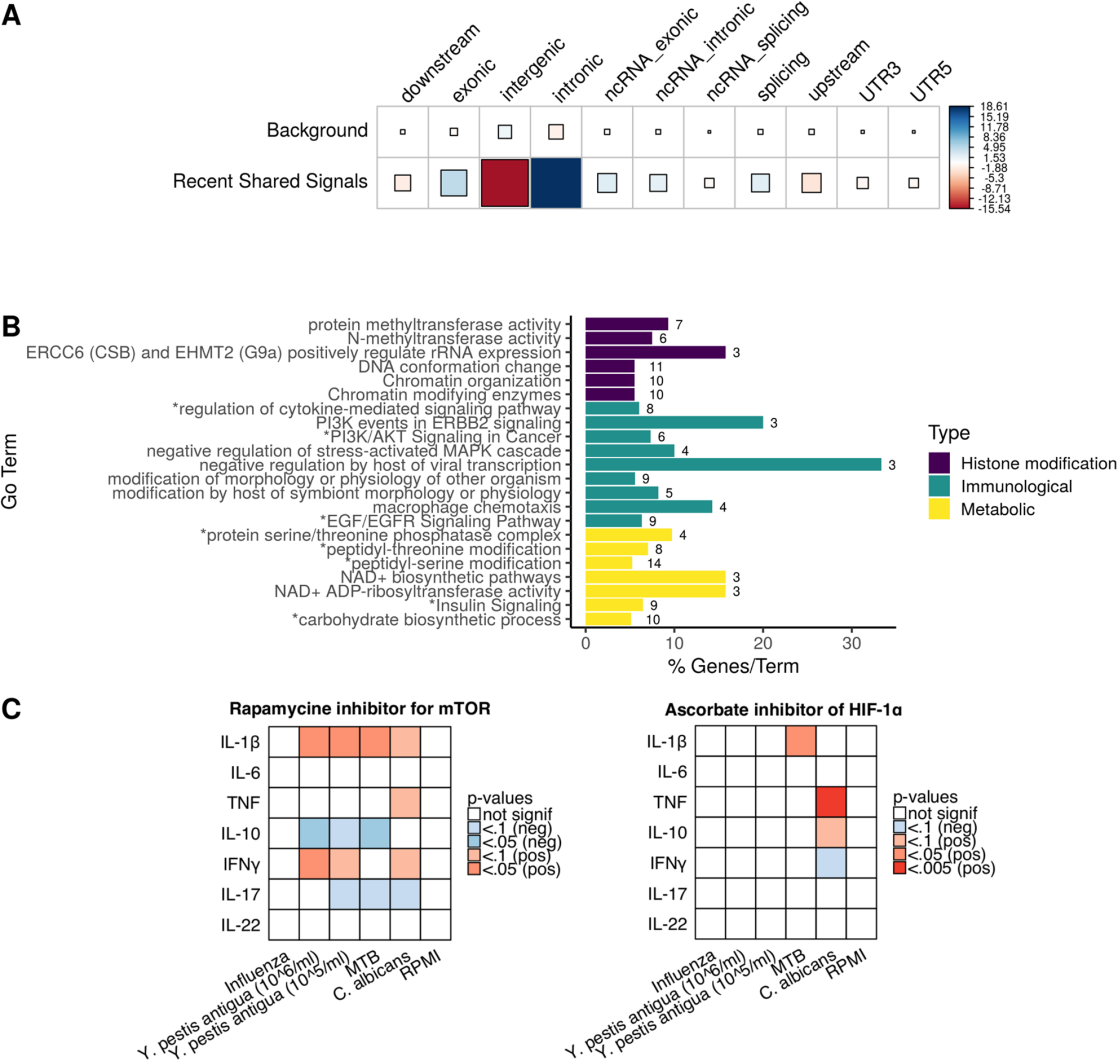
Genomic distribution, local ancestry, and enrichment analysis of the selection signals. (**A**). Enrichment of SNP functional categories. Square size is proportional to the contribution of each SNPs functional category to the total χ^2^ score indicated by the Pearson residuals. Positive values (in blue) indicate that the proportion of that category is higher than expected in the signals whereas negative values (in red) indicate that that signals are depleted in that functional category. (**B**) Pathway enrichment analysis based on the genes under selection. Pathways with an excess of European local ancestry are marked with an asterisk (p-value < 0.05, Benjamini and Hochberg—False Discovery Rate (BH—FDR)). Bar length is proportional to the percentage of genes in the term found within our signals, while the number of genes found in each term is shown. Pathways are colored depending on their main biological function. All terms are statistically significant (p-value < 0.05, BH—FDR). (**C**) Modulation of cytokine production by PI3K-mTOR-HIF-1α. Heatmap of cytokine production after pathogen stimulation and inhibition of mTOR (by Rapamycine) or HIF-1α (by ascorbate). Significant p-values are indicated with colors: red indicates an increase in cytokine production after inhibition, whereas blue indicates a decrease. P-values were corrected for multiple testing by FDR.

These selection signals comprise a total of 28,640 SNPs, among which we found more SNPs located in genic regions (intronic and exonic) than expected by comparing with a genome-wide distribution (Pearson's Chi-squared test, χ^2^ = 632.89, 10 df, p-value < 1e-16) (Fig. 3A). Overall, we observed higher local European ancestry in the regions under selection (78.28 ± 9.7%, mean ± sd) than the genome-wide average (Permutation test 10,000 permutations, p-value < 2.2e-16).

When looking for functional candidate variants, out of the 28,640 SNPs under selection, 6,452 are highly differentiated when comparing the two populations sharing these recent signals (Roma and non-Roma Romanians) to Northwest India (see “Methods”). Of those, 293 were predicted to be among the 10% most deleterious changes in the human genome (i.e. CADD values ≥ 10), 28 were non-synonymous changes (including 9 SNPs with CADD values ≥ 10), 18 implied synonymous changes, and one was a stop gain change (with a CADD value = 40) (Supplementary Table 7). Out of the 16 candidate genes with highly differentiated non-synonymous or stop gain variants, two candidate genes are related to the immune system: *ELF1* (E74-Like Factor 1), which is expressed in lymphoid cells and participates in the T-cell-receptor-mediated trans activation of HIV-2 gene expression^35^; and *SETX*, which participates in controlling antiviral response^36^. Notably, several of the non-synonymous variants linked to the selection signals were found associated with bone mineral density: rs2287679 and rs10416265 in *GPATCH1*^37–40^ and rs11917356, rs12488457 (with a CADD value = 23), rs16827497, and rs1497312 in *COL6A5*^41^.

Since in the genic regions the enrichment of SNPs under recent selection was strikingly higher in introns than in exonic sequences (Fig. 3A), the most interesting functional candidate variants under selection may relate to the regulation of those genic regions rather than to non-synonymous changes in exons.

### Recent shared signals are enriched in pathways implicated in cell metabolism rewiring during immune stimulation

To identify the selective pressures faced by the Roma in their new environment, we performed an enrichment pathway analysis on the genes identified in the selection signals (Fig. 3B, Supplementary Fig. 12). Several of the pathways are related to housekeeping processes, involved in functions that cannot be linked to specific phenotypes that could be at the base of the action of selection, such as cytosolic transport or Golgi cisterna membrane (Supplementary Fig. 12). This result seems to follow the omnigenic model^42^, in which the strong interconnection among the gene regulation networks might result in signals (of susceptibility in GWAS studies, of positive selection in genome scans) that are not of direct relevance for the selected phenotype.

However, several important immune regulatory, metabolic and histone modification pathways have been identified to be under shared recent selection (Fig. 3B). Out of the 22 pathways overrepresented in the selection signals, eight of them also present an excess of SNPs with high European ancestry (p-value < 0.05 after multiple testing correction by FDR). Rewiring of cellular metabolism has been recently described to be an important component of the response of immune cells^43–45^. The shift from oxidative phosphorylation to aerobic glycolysis (“Warburg effect”) is needed to fulfill the energy requirements of clonal expansion in activated lymphocytes during the process of antigen presentation by myeloid cells. The shift towards aerobic glycolysis by activated immune cells implies increased glucose consumption and the reduction of NAD^-^ to NADH, as well as epigenetic changes in glycolysis-related genes, and it is mediated, among others, by the PIK3-mTOR-HIF-1α pathway^43,46^. All these biological processes were found enriched in the selection signals (Fig. 3B), and we validated the role of the PIK3-mTOR-HIF-1α pathway for cytokine production capacity induced by pathogens known to have contributed to selective pressures in populations in Europe: *Yersinia pestis* antigua (the agent of plague), influenza virus, *Mycobacterium tuberculosis* (MTB), and *Candida albicans* as the most common human fungal pathogen^18,47^.

Our results show that inhibition of mTOR pathway generates a cytokine profile characterized by a decrease of IL-10 and IL-17, and an increase of IL-1β (Fig. 3C), supporting the idea that mTOR pathway modulates cytokine production in immune cells^48^. This effect is exerted by both bacterial and fungal stimuli, but not by influenza. On the other hand, even though multiple human pathogens induce activation of HIF-1α^49^, its inhibition influenced only *Candida-* and MTB-induced cytokine responses. HIF-1α inhibition increased TNF and IL-10 levels after *Candida* infection, and it has been previously shown that HIF-1α controls progression of fungal infections by limiting IL-10 production^50^. HIF-1α mediated glycolytic pathway acts downstream of mTOR, which might explain why the inhibition of PIK3-mTOR-HIF-1α pathway at different levels generates an opposite reaction in INF-γ: mTOR inhibition increases INF-γ production after *C. albicans* stimulation, whereas inhibition at the level of HIF-1α reduces its production.

### Recent shared signals are enriched in cytokine QTLs (cQTLs)

**Figure 4:**
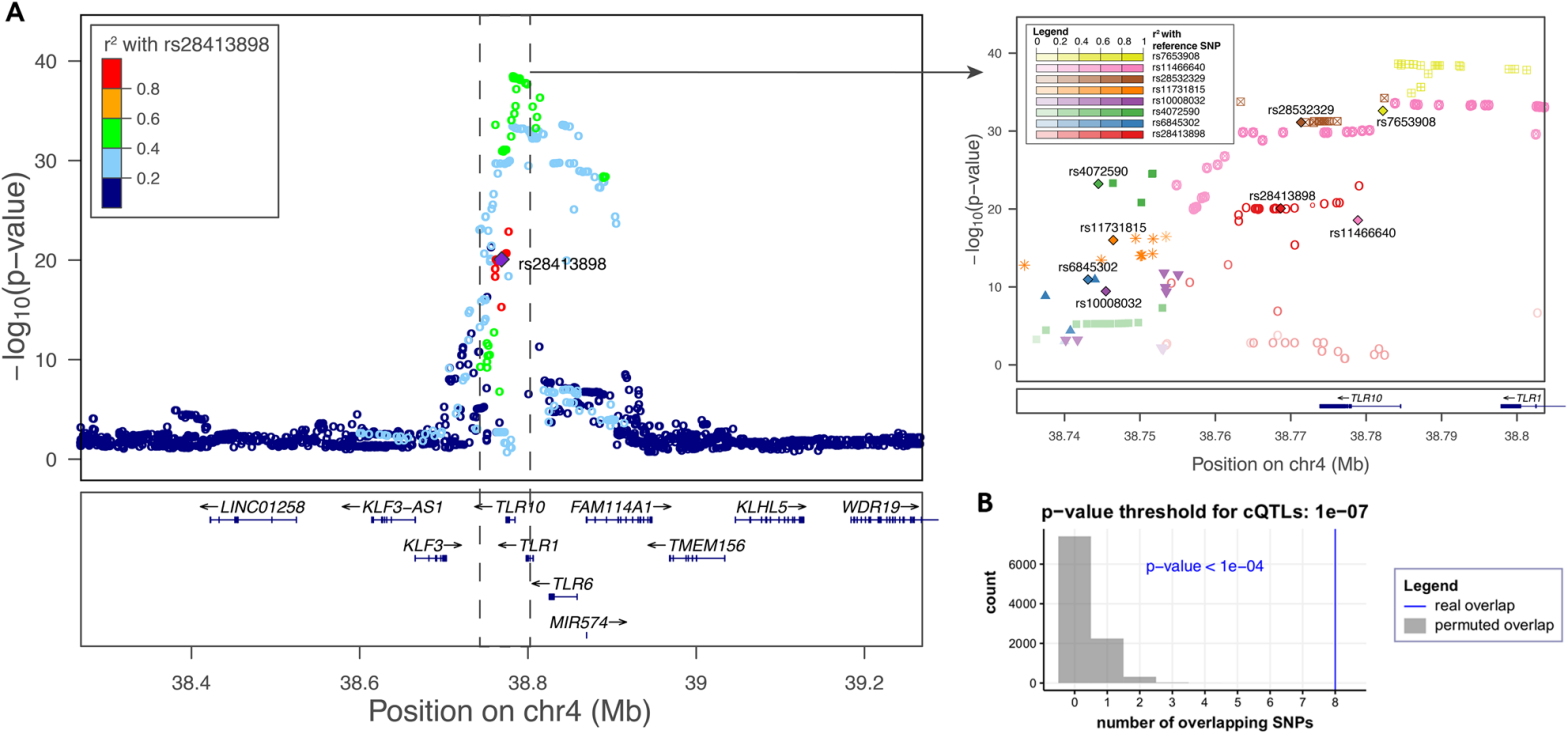
Enrichment of cQTLs in the selection signals. (**A**) Local Manhattan plot of the region in which the 8 cQTLs found in the selection signals are located. The left plot shows a region of 1 Mb, with R^2^ values calculated relative to rs28413898 (selected as reference point as it is in the center of the region). The right plot shows a zoomed in region of 70 Kb, where for each of the 8 SNPs the other SNPs in the same region most strongly in linkage disequilibrium are shown in the same color scheme. Both plots were created using LocusZoom^74^. LD-values are based on hg19 EUR 1000 Genomes 2014. (**B**) Number of cQTLs found in the selection signals (blue line) compared to permutation results (grey histogram). Overlap between the top cytokine QTLs (cQTLs, p-value < 1e-7) and pruned selection signals. The overlap was compared to 10,000 random permutations. cQTL results for different stimuli and cytokines were merged by taking the lowest p-value for each SNP.

Among the immune pathways identified to be under recent selection both in Roma and non-Roma Romanian populations that have an excess of SNPs with local European ancestry, regulation of cytokine signaling is a central initial step for activation of host defense. The cytokine network is the main regulatory system in inflammation and host defense, and due to its importance, we aimed to experimentally validate its evolutionary relevance. Using the data from the 500FG cohort from the Human Functional Genomics Project^51^, we assessed whether there is an enrichment of Quantitative Trait Loci (QTLs) influencing cytokine production capacity (cytokine QTLs or cQTL) in the selection signals. Recent selection in Roma and non-Roma Romanians targeted genetic variants that affect the expression of cytokines (Fig. 4). A total of 8 SNPs had an overlap between the selection signals and cQTLs (p-value < 1e-7) (Supplementary Table 8). Moreover, 7 out of these 8 SNPs had cQTL p-values smaller than 1e-10 (Supplementary Fig. 13). Specifically, these 8 SNPs are in the *TLR1/6/10* region. The *TLR1/6/10* region has been identified as target of positive selection using individuals of the same populations^18^ and recently shown to have the strongest influence on cytokine production capacity^51^.

We have also assessed whether the 28 highly differentiated non-synonymous SNPs on the candidate genes identified in the selection signals also influence cytokine production capacity. Among those, two *GPATCH1* polymorphisms (rs2287679 and rs10416265), consistently influenced cytokine production induced by influenza, suggesting a new biological role for *GPATCH1* in antiviral immunity. While little is known about its immunological function, recent studies have suggested that Ecgp96, the protein encoded by *GPATCH1*, regulates the pathogen recognition by TLR2 during bacterial infection^52^, which may contribute to the regulation of cytokine production.

## Discussion

In the present study we analyzed for the first time whole-genome sequences of Roma individuals and compared them with individuals of European ancestry (Romanians) living in close geographic proximity, and the putative source of Roma population from Northwest India. We show that strong selective pressures have been exerted on immunological and metabolic processes in both the genome of the Roma and non-Roma Romanians (and not in Indians) during the last thousand years of shared history.

Upon migration in new geographical areas, humans are sometimes exposed to radically different infections, leading to strong changes and adaptation in immune responses^53^. Subsequently, populations of different genetic backgrounds that have shared similar environments have been forced to undergo similar evolutionary changes in their immune defenses. This led to select the same genomic regions with genetic information useful for immune host defense in both populations, something that has happened between archaic and modern humans^54,55^, and in populations living in the same geographic areas in East Africa^56^ or Europe^18^. In this study, we performed an in-depth analysis of the shared evolutionary changes in two populations of different genetic background (European/Romanian vs Roma) living in the same area of South-East Europe. Roma arrived in Romania in the fourteenth century^1,4^. Despite their origin in the Indian subcontinent^2,12,14,15^, they are genetically more similar to other Central and South Eastern European populations than current Northwestern Indians populations (Fig. 1B). This could be explained by their complex demography with a series of bottlenecks and a high consanguinity in the Roma (Fig. 2, Supplementary Fig. 10), that would have increased the genetic drift in this population, together with an important admixture with the host European populations^2,11,14,16^.

We used XP-EHH specifically to detect recent strong selection (complete selective sweeps) in both Roma and non-Roma, as XP-EHH is not affected by genome-wide differences in haplotype length between populations with different demographic histories^32^. Despite the different origin and demographic history compared to European Romanians (Fig. 2, Supplementary Fig. 12), Romanian Roma have between 48–54% of West Eurasian ancestry (Supplementary table 6). Deviations from such genome-wide ancestry proportions in a locus can help to detect beneficial alleles introduced by admixture. If an allele is at high frequency in an European population and the proportion of European-like admixture in the Roma is ~ 50%, for the allele to be detected under a complete selective sweep in the Roma, its frequency should increase from 50% to 80–100%. After the initial admixture event, there are two processes that can spread the allele to the rest of the Roma population: random drift or selection. If a complex phenotype is under selection, there will be several functionally related alleles/genes that are selected and whose frequency will increase in the population. In the case of random drift, some alleles will increase in frequency while others will decrease, even if they are functionally related. Eight of the pathways overrepresented in the selection signals related to immune and metabolic processes also have an excess of SNPs with high European ancestry (p-value < 0.05 after multiple testing correction by FDR), including the regulation of cytokine mediated signaling pathway. We hypothesize that some of the regions found under selection in both the Roma and the non-Roma Romanians were either under selection in non-Roma and continued under selection in the two populations after being introduced by admixture in the Roma (adaptive admixture), or that they were selected post-admixture in both populations due to a shared selective pressure. These deviations from local ancestry in specific pathways suggest that adaptive admixture has played a role in shaping the adaptation of the immune system in the Roma after the diaspora.

We identify numerous and strong common selective signals in the two populations for recent times, such as immune response and cellular metabolism (Fig. 3B), that very likely contributed to the fitness of the populations during the common selective pressures of the last millennium (e.g. plague, tuberculosis or influenza epidemics). Among the immunological pathways identified to have been under selection, cytokine signaling is a very relevant immune pathway. Cytokines are the main communication system within the immune system, and selective pressures on several major groups of genes influencing these responses such as TLRs and interferons have been identified^57^. Our data strongly support the concept that cytokine responses have been under strong evolutionary pressures (Fig. 4). Moreover, our analyses also identify important specific patterns of interaction between one of the most important metabolic signaling pathways (PIK3-mTOR-HIF-1α) and cytokine responses (Fig. 3C). In this respect, mTOR mainly influenced bacterial and fungal induction of cytokines, whereas viral (influenza) stimulated cytokines were not impacted. HIF-1α is one of the transcription factors activated by mTOR that has an important role in stimulation of glycolysis^45,46^: interestingly its inhibition mainly influenced fungal stimulation of cytokines. This argues that mTOR acts on fungal regulation of cytokines through HIF-1α, while other pathways must be involved in the mTOR effects on bacterial stimulation of cytokine production.

In conclusion, these data help us to discern the broad picture of the evolutionary processes that have shaped Roma and non-Roma Romanian populations living in Europe side-by-side during the last millennium, arguing for important evolutionary processes that have influenced their genomes. Moreover, the identification of the immune pathways under selective pressures contributes to the description of important patterns in the response to bacterial, viral and fungal pathogens, and leads to an improved understanding of the host defense against infectious disease.

## Methods

### Samples

All procedures performed were in accordance with the ethical standards of, and approved by, the Ethics Committee of the University of Craiova, Romania. After approval, informed consent was obtained for all volunteers. DNA samples were collected from 50 individuals of European/Romanian descent, and 50 individuals of Roma (Romani/Rroma) ethnic background, all from South-West of the country (Dolj County). We generated whole genome sequences with an average of 15 × coverage (see Supplementary Note 1 for technical details). After strict quality control (Supplementary Note 2–3, Supplementary Figs. 1–6, Supplementary Tables 2–3), we were left with 40 unrelated non-Roma (RMN) and 40 unrelated Roma (ROM) for the main analyses (see Supplementary Table 1 for reasons of exclusion). For each newly sequenced sample, the mitochondrial consensus sequence constructed for the estimation of mitochondrial contamination was used to identify their mitochondrial haplotype using mt-classifier from MToolBox^58^.

### Population demographic analyses

We combined the new data generated in this study with populations covering the genetic diversity present on continental India^59^ and worldwide populations^60,61^ (Supplementary Note 1 and 3 for population codes). We filtered the data with PLINK 1.9^62^ to keep only bi-allelic autosomal SNPs with Minor Allele Frequency (MAF) > 0.05, under Hardy–Weinberg Equilibrium (p-value ≥ 0.000001) and without missing data, obtaining a dataset with 938 samples and 5,216,078 SNPs. This dataset was pruned for linkage disequilibrium (LD) in 515,723 SNPs. We performed a principal component analysis (PCA) with EIGENSOFT v6.1^63^ on the pruned dataset without the African populations. Runs of homozygosity (ROH) were estimated by PLINK 1.9 with arguments –homozyg-snp 100 –homozyg-window-het 1 –homozyg-kb 1000 leaving the rest of parameters as default.

The admixture analysis with ADMIXTURE v1.23^64^ was run with values of K ranging from 2 to 9, each K was run 25 times with fivefold cross-validation and different seed to estimate the best supported model in the pruned dataset.

Local and global ancestry inference was estimated by RFMix (version 1.5.4)^65^ on the phased dataset without missing data following the pipeline suggested by Martin et al.^66^ (https://github.com/armartin/ancestry_pipeline). RFMix PopPhased was run using 3 expectation–maximization (EM) iterations, window size of 0.2 cM, with a minimum number of reference haplotypes per tree node of 5 as we have an unequal sample size for the reference populations. Time since the admixture between the two populations was set at 25 generations ago. The rest of parameters were set with default values. Non-Roma Romanian were used as the putative European source (n = 40) and Rajput (RAJ) as the putative North Indian source (n = 10) of the Roma genome. We only considered sites assigned to one ancestry with a probability higher than 99%. We calculated the shared genetic drift by outgroup f3 statistic in the form f3(ROM, X; African (YRI)), where X is a European, a South East Asian population, or the newly sequenced Romanian population. To test whether Roma are an admixed population, we used the D-statistics in the form of D(European, African(YRI), ROM, South Asian), and estimated the proportion of West Eurasian ancestry in the Roma using the f4 ratio estimation in the form of α = f4(African(YRI), European; ROM, RMN)/f4(African(YRI), European, RMN, RAJ). F-statistics based analyses were performed as implemented in ADMIXTOLS^20^ with the R package admixr^67^.

To estimate Roma and non-Roma Romanian recent demographic history, we detected identity-by-descent (IBD) segments using IBDseq (version r1206) with default parameters^68^. Then, the variation of population effective size (Ne) through time was estimated with IBDNe (version 19Sep19.268)^31^. IBDNe uses long IBD segments (> 2 Mb) to infer Ne values with 95% confidence interval (CI) at each generation (generation time = 25 years).

### Scan of selection

The objective of this analysis was to identify the common selective pressures that both European non-Roma and Roma populations faced after the latter settled in Romania. We used the Cross-population Extended Haplotype Homozygosity (XP-EHH) test^32^ computed pairwise to detect signals of recent positive selection that are shared by these two populations but not a third population of Northwest Indians, used as surrogate of the original proto-Roma population. XP-EHH normalizes for genome-wide differences in haplotype length between populations with different demographic histories (Roma and non-Roma Romanians)^32^, and our pairwise approach allows further reduction of false positives by filtering out regions that are not selected in both populations when comparing with a third one. XP-EHH was run as implemented in selscan^33^ after phasing the data. Each population was phased separately with SHAPEIT2^69^ with default parameters and using the 1,000 Genomes Phase 3 panel of haplotypes^60^ as a reference dataset. SNPs with missing data were removed. We obtained the genetic position and ancestral allele information from the 1000 Genomes Project^60^.

We analyzed 40 Roma (ROM) and 40 non-Roma Romanians (RMN) and 10 Rajput (RAJ)^59^ individuals. We selected the Rajput as the best proxy for the Indian population more related to the proto-Roma because they are located in Northwest India, closest to the putative area of origin of the Roma people (based on Y-chromosome markers^13^ and autosomal SNPs^2,15^). To minimize biases due to sequencing technologies or variant calling algorithms, the sequences of the three populations were obtained within the same project. XP-EHH was run using default parameters, only reducing the maximum allowed gap between two SNPs from 200,000 to 20,000 bp to avoid spurious peaks. We performed three comparisons: Roma vs. non-Roma Romanian, Roma vs. Northwest India, and non-Roma Romanian vs. Northwest India. We calculated the average value of XP-EHH in 30 kb windows with an overlap of 5 kb.

We selected the windows shared between the 5% upper tail genome-wide distribution of the non-Roma Romanian vs. Northwest India and Roma vs. Northwest India comparisons, keeping SNPs with XP-EHH > 2 in the Romanian populations. From those we removed the markers belonging to the windows in the 5% upper and lower tail of the Roma vs. non-Roma Romanian comparison, to minimize the false positives. The remaining regions correspond to the genomic regions under positive selection in both Roma and non-Roma Romanians, and not in Northwest India and therefore, they would indicate the selective pressures Roma people faced when they established themselves in Romania.

The regions under positive selection were annotated with ANNOVAR^70^ in GRCh37 (hg19) using RefSeqGene, dbSNP 147, and CADD (Combined Annotation Dependent Depletion) version 13^71^. We computed derived allele frequency (DAF) differences between Roma and Northwest India and between non-Roma Romanians and Northwest India, and variants were classified as highly differentiated when the average DAF difference to Northwest India was greater than 0.25. Subsequently, those highly differentiated SNPs that are either non-synonymous, annotated as cis-eQTLs in the Genotype-Tissue Expression Project (Release V6p), present CADD values greater than 10 (meaning they are predicted to be among the 10% most deleterious in the human genome), or that appear clustered in exonic/splicing regions/ncRNAs/UTRs were classified as potential candidate variants for adaptation.

Finally, we also performed a two-sided Gene Ontology (GO) enrichment analyses (Enrichment/Depletion) and pathway annotation network tests with Cytoscape^72^ plug-in ClueGo^73^ in the selection signals. P-values were corrected for multiple testing by the Benjamini-Hochberg (BH) procedure.

We compared the average proportion of local European ancestry in the selection signals to the sampling distribution of the mean genome-wide proportion of European ancestry estimated by a permutation test (10,000 permutations). We also tested for an excess of local European ancestry in the pathways found overrepresented in the selection signals. We calculated the 95th percentile of the European ancestry in the Roma using all SNPs located in genes, excluding the genes detected under positive selection. Then, for each pathway, we calculated the proportion of SNPs belonging to the genes in that pathway with higher European ancestry than the 95th percentile. To test whether there was an excess of SNPs with high European ancestry in a given pathway, we repeated this process 1,000 times by randomly sampling from the whole genome as many genic SNPs as SNPs are in the pathway and calculating the proportion of SNPs with higher European ancestry than the 95th percentile. P-values were corrected for multiple testing by FDR.

### Functional validation of pathways under positive selection: modulation of cytokine production by PIK3-mTOR-HIF-1α

To functionally validate the role of PI3K-mTOR-HIF-1α signaling pathway on immune response, we tested whether its inhibition affected the cytokine production capacity of peripheral blood mononuclear cells (PBMC) stimulated with different pathogens (Supplementary Note 4). We inhibited the PI3K-mTOR-HIF-1α pathway with Rapamycine (mTOR inhibitor, 10 nM) and Ascorbate (HIF-1α inhibitor, 50 µM), based on earlier studies in our laboratory which tested the optimal experimental conditions^46^ and tested a total of 5 pathogens using RPMI as negative control: *Yersinia pestis* antigua (10^6^/ml); *Y. pestis* antigua (10^5^/ml); Influenza (× 10); *Mycobacterium tuberculosis* (MTB, 5 µg/ml); and *Candida albicans* (10^6^/ml). For each stimulation we measured the expression levels of 7 cytokines: IL-1β, IL-6, TNF, IL-10, IFNγ, IL-17 and IL-22. These were all measured for a total of 8 subjects (3 separate experiments). Differences were assessed by permutations within batches (all possible permutations [permutations = 172,800], paired t-test, two-sided p-value).

### Functional validation of pathways under positive selection: cytokine regulation

To validate the link between the regulation of cytokine-mediated signaling pathway and the signals of positive selection, we assessed whether there is enrichment of Quantitative Trait Loci (QTLs) influencing cytokine production capacity (cytokine QTLs or cQTL) within those signals of positive selection.

*Cytokine stimulation in 500 Functional Genomics (500FG) cohort.* We used the results of cytokine production (after 24 h for TNF, IL-1β and IL-6 stimulation and after 7 days for IFNγ, IL-17 and IL-22) under different stimuli (bacterial, viral and fungal) obtained from the 500FG cohort of healthy individuals of European ancestry from the Human Functional Genomics Project^51^. As described in Li et al.^51^, genotype data and cytokine production capacity data was available for 442 individuals.

*Cytokine enrichment analysis:* We assessed whether there was an enrichment of cQTLs in the selection signals by a randomization test. We analyzed four sets of SNPs: (I) All SNPs considered in the cQTL dataset^51^ (from now on referred to as cQTL background, n = 4,358,038 SNPs), (II) all SNPs considered in the selection analysis (from now on referred to as selection background, n = 4,644,113 SNPs), (III) the SNPs with p-values < 1e-7 in the cQTL dataset (from now on referred to as cQTL significant, n = 335 cQTLs), and (IV) the SNPs in the top 5% windows in the selection analysis after LD pruning (from now on referred to as selection top 5%, n = 3,382 SNPs).

In this analysis we only consider SNPs that are present in both background sets (3,572,470 SNPs). The randomization is performed as follows: first the selection top 5% is pruned down to only contain SNPs that are not in LD. LD was defined as R^2^ >  = 0.8 and calculated based on the genetic data used for cQTL analysis, and SNPs had to be within 1 Mb. Pruning was performed keeping the higher selection scores for a pair of SNPs in linkage disequilibrium. The real overlap is calculated between the selection top 5% and the cQTL significant SNPs. Following this, 10,000 random permutations are performed in which X SNPs are selected from the selection background, where X is the size of the selection top 5%. We count the number of times the permutated overlap is equal to or higher than the real overlap (which we name “S”), and calculate a p-value using the following formula:

$$p-value\le \frac{1+S}{number\_of\_permutations}$$

To validate robustness against p-value threshold for the cQTLs and the outlier approach for the selection signals, the procedure was repeated for different thresholds (p-value < 1e-10, 1e-8, 1e-7, 1e-6, 1e-5, 1e-4) and chosen percentages (2% and 5%; Supplementary Fig. 13). Up until a p-value cut-off of 1e-4, the results were comparable.

## Supplementary information

Supplementary file1

## Data Availability

Genome data generated in this study (BAM and VCF files) has been deposited at the European Genome-phenome Archive (EGA) which is hosted at the EBI and the CRG, under Accession Number EGAS00001003624.
