# Supplementary material for: The shaping of immunological responses through natural selection after the Roma Diaspora
Source: Sci Rep. 2020 Sep 30;10:16134. doi: 10.1038/s41598-020-73182-1 (PMC7528012; doi:10.1038/s41598-020-73182-1)
Supplement: Supplementary file 1 — Supplementary file1 [file 41598_2020_73182_MOESM1_ESM.pdf]

Supplementary Information: The shaping of immunological responses through natural selection after the Roma Diaspora

Begoña Dobon[1#†], Rob ter Horst[2#‡], Hafid Laayouni[1,3], Mayukh Mondal[4], Erica Bianco[1], David Comas[1], Mihai Ioana[5], Elena Bosch[1,6], Jaume Bertranpetit[1*], Mihai G. Netea[2,5,7*]

[1]Institut de Biologia Evolutiva (UPF-CSIC), Universitat Pompeu Fabra, Doctor Aiguader 88 (PRBB), 08003 Barcelona, Catalonia, Spain.

[2]Department of Internal Medicine and Radboud Center for Infectious Diseases, Radboud University Medical Center, 6525 GA Nijmegen, the Netherlands.

[3]Bioinformatics Studies, ESCI-UPF, Pg. Pujades 1, 08003 Barcelona, Catalonia, Spain

[4]Institute of Genomics, University of Tartu, Tartu, Estonia

[5]Department of Human Genetics, University of Medicine and Pharmacy Craiova, Romania

[6]Centro de Investigación Biomédica en Red de Salud Mental (CIBERSAM), 43206 Reus, Spain

[7]Department for Genomics & Immunoregulation, Life and Medical Sciences Institute (LIMES), University of Bonn, 53115 Bonn, Germany.

# equal contribution

† Current affiliation: Department of Anthropology, University of Zurich, Zurich, Switzerland

‡ Current affiliation: CeMM Research Center for Molecular Medicine of the Austrian Academy of Sciences, Vienna, Austria.

*Corresponding authors: jaume.bertranpetit@upf.edu or mihai.netea@radboudumc.nl

Supplementary Note 1. Data preprocessing

Samples

We generated whole genome sequences of 50 Roma and 50 non-Roma Romanian individuals from the Dolj County (South-West Romania). To study the ancestry of Roma people and avoid any ascertainment bias in the variant calling (process described in Raw Sequence Processing and Mapping) we included whole genome sequences of a set of Indian populations that reflect the maximum genetic diversity on continental India[1]: 10 Uttar Pradesh Upper Caste Brahmins (UBR), 10 Rajput (RAJ), 10 Irula (ILA), 10 Riang (RIA), nine Birhor (BIR), nine Vellalar (VLR), one Punjabi (PUN),

one Bengali (BEN), six Onge (ONG) and four Jarawa (JAR). We also included one individual from each of the following populations to have a worldwide representation of human genetic diversity: French (FRN), Sardinian (SAR), Dai (DAI), Han Chinese (HAN), Mandenka (MAD), Mbuti (MBT), Papuan (PAP), San (SAN), and Yoruba (YRI)[2].

Library preparations, sequencing, and base calling

DNA was extracted from blood samples and it was sequenced at the Beijing Genomics Institute (BGI; Beijing, China). For every sample, 1 µg of genomic DNA was sheared into short fragments on Covaris E210 system (CovarisInc). The overhang at the ends of DNA fragments were converted into blunt ends by T4 DNA polymerase and Klenow enzyme. After ligation with adapters on both ends, DNA fragments of ~500 bp were selected by agarose gel electrophoresis and purified. Polymerase Chain Reaction (PCR) was performed to obtain enough DNA for a sequencing library. The quality of the library was checked by agarose gel electrophoresis. Sequencing was performed on Illumina HiSeq 2000 to produce paired-end reads of 90 bp. Base calling was completed following the manufacturer's base-calling pipeline.

Raw Sequence Processing and Mapping

Fastq conversion, mapping and BAM processing was performed following a previously described procedure (Supplementary material)[1].

- **Fastq Conversion**: Sequences from BGI were in Illumina 1.5+ FASTQ format. All the BGI FASTQ files were converted to Illumina 1.8+ using seqtk () with the -VQ64 flag (./seqtkseq -VQ64). FASTQ files from human populations from Meyer *et. al*[2] and from Indian populations from Mondal et. al[1] were downloaded and converted to Illumina 1.8+ format using seqtk. The following steps (Mapping, BAM processing and Variant Calling) were applied to all sequences of Roma and Romanian populations, along with sequences from Indian and worldwide datasets.

- **Mapping:** All sequences, in Illumina 1.8+ FASTQ format, were mapped using BWA[3]. Hg19 was used as a reference and mapped using the BWA mem algorithm. Only paired-end reads were kept. The BWA -w 50 flag was used to

give the size of the band width. BWA output was then converted to binary format (bam) using SAMtools (version 0.1.18)[4] and sorted using SortSam from Picard tools (version 1.100).

- **BAM Processing:** Bam processing was completed by following the "Best Practices" recommendations in GATK (version 3.5)[5] . After converting the mapped files to the binary format, CleanSam from Picard tools (http://picard.sourceforge.net) was used to remove unmapped sequences, and MarkDuplicates to mark duplicates. The bam files were then indexed using SAMtools. Since indels can cause inaccurate mapping in the genome, IndelRealigner from GATK was used to realign them, with 1000 Genomes Project Phase 1 Indel as a reference file (interval file)[6]. BaseRecalibrator and PrintReads were used from GATK to calibrate bases for various statistics (i.e. reported quality score, machine cycle, positions of the SNP in the read, etc.) for SNPs not present in dbSNP version 137[7]. MergeSamFiles from Picard tools was used to merge lanes for the same individuals before variant calling by GATK.

- **Variant Calling:** Variant calling for Roma and non-Roma Romanian sequences, along with Indian and worldwide sequences, were done by GATK. Per-sample calling was done with default options and using -- max-alternate_alleles 20 (to capture all genetic diversity present in the populations) by running HaplotypeCaller in GVCF mode on each sample's BAM file. Then, the joint genotyping of the gVCFs produced was done by running GenotypeGVCFs on all of them together to generate a raw SNP and indel Variant Calling File (VCF).

- **VCF Recalibration:** The raw VCF was filtered using post variant calling recalibration steps as listed in GATK "Best Practices". VariantRecalibration and ApplyRecalibration from GATK were used to calculate various statistics for novel variants (both for SNPs and indels) and then recalibrated according to their needs. We applied the following steps:

    - SNPs with the flags -an QD -an MQRankSum -anReadPosRankSum -an FS -an DP -an InbreedingCoeff. All other parameters were set to default values:

    - dbsnp version 137: -resource:dbsnp, known=true, training=false, truth=false, prior=2.0.
    - hapmap version 3.3: -resource:hapmap, known=false, training=true, truth=true, prior=15.0.
    - Omni genotyping array 2.5 million 1000G: -resource:omni, known=false, training=true, truth=true, prior=12.0.
    - 1000G phase 1 high confidence: -resource:1000G, known=false, training=true, truth=false, prior=10.0.
- Indels with the flags -- maxGaussians 4 -an FS -an ReadPosRankSum -an MQRankSum -an DP -an InbreedingCoeff. All other parameters were set to default values:
    - Mills 1000G high confidence indels: -resource:mills, known=false, training=true, truth=true, prior=12.0.
    - dbSNP version 137: -resource:dbsnp, known=true, training=false, truth=false, prior=2.0.

Supplementary Note 2. Quality control

Depth of Coverage and Fraction Covered

Genome coverage for each sample was estimated by DepthOfCoverage from GATK to check for bias in the probability of calling non-reference alleles due to different coverage between samples. The average coverage for autosomal chromosomes ranged from 12X to 21X, with an average of 15X (Supplementary Figure 1).

Sex determination

We also estimated the coverage for the X and Y chromosomes to determine the genetic sex of the samples by DepthOfCoverage from GATK. We calculate the ratio of the coverage on the X and Y chromosomes with respect to the coverage on autosomal chromosomes. In females, we expect the ratio of the coverage on the X chromosome and the coverage on the autosomes to be around one; whereas in males, it should tend to 0.5 (males only have one copy of the X). In males, we expect the ratio of the coverage on the Y chromosome and the coverage on the autosomes to tend to 0.5, whereas it should be zero in females (males only have one copy of the Y, and females none).

We observed a sample with ambiguous sex determination, sample RMN-17 (Supplementary Figure 2). This can indicate contamination of the sample and was further analyzed in the Estimation of heterozygosity and mitochondrial contamination sections. Four non-Roma Romanian samples were identified as female (RMN-7, RMN-12, RMN-14, and RMN-31), the rest were classified as male. All Roma samples were classified as male.

Estimation of autosomal heterozygosity (inbreeding)

The inbreeding coefficient (F) was calculated for each sample by VCFtools (version 0.1.14)[8]. Individuals showing an outlier value of heterozygosity or F could be the result of contamination. Sample RMN-17 showed an extremely low value of F compared to any other sample (Supplementary Figure 3) and was removed from the main analysis (Supplementary Table 1).

Estimation of heterozygosity in males (X chromosome)

We estimated contamination levels based on the level of X-chromosome heterozygosity in male samples with ANGSD[9]. As requested by the software we used a list of polymorphic sites and their frequency for the following populations from 1000 Genomes Project: CEU (Utah Residents (CEPH) with Northern and Western European Ancestry), CHB (Han Chinese in Bejing, China), PEL (Peruvians from Lima, Peru), YRI (Yoruba in Ibadan, Nigeria), and GHI (Gujarati Indian from Houston, Texas). As a recommendation, samples with X chromosome contamination estimates higher than 2.5% should be classified as contaminated. Only RMN-17 appears affected with a 33-43% of contamination (Supplementary Table 2) and was removed from the main analysis (Supplementary Table 1).

Estimation of mtDNA contamination

Estimation of mitochondrial genome contamination was done by Rpackage contamMix (version 1.0-10)[10,11] to identify mitochondrial heteroplasmy. First, a mitochondrial consensus sequence was constructed for each sample with SAMtools mpileup (version 1.2) filtering for reads with excessive mismatches (-C 50), minimum mapping quality (-q 20) and minimum base quality for a base (-Q 20). Then, the mitochondrial reads were mapped against the mitochondrial consensus sequence using BWA with the -w 50 flag (size of the band width) and the output converted to bam format. Second, we generated a multiple sequence alignment with the consensus genome and the 311potential contaminant mitochondrial genomes provided in contamMix package using Muscle (version 3.8.31)[12]. With these two inputs, the program estimates the proportion of endogenous (authentic) mitochondrial genome present in the sample (P.AUT). A P.AUT of 0.80 means there is 20% of contamination. A sample was classified as possibly contaminated if the proportion of reads that have a better match with the consensus sequence generated than with any of the 311 mitochondrial sequence provided is less than 95%, or if the 95% confidence lower bound of that proportion is less than 85%. There are 5 samples showing more than 5% of contamination in the mitochondrial genome (Supplementary Figure 4) even though only RMN-17 showed any sign of contamination in the other analysis. This could be due to different amplification of autosomal and mitochondrial reads, and samples RMN-17, S19, S25, S43, and S60 were flagged as contaminated. This analysis was repeated adding the mitochondrial consensus sequence of RMN-17 to the set of 311 potential contaminant mitochondrial genomes as a potential source of contamination with the same results

(data not shown). Samples marked as contaminated were removed from the main analysis (Supplementary Table 1).

Transition versus Transversion Ratio

The transition vs. transversion ratio (Ts/Tv) can indicate whether there are problems with the variant calling of the samples. VariantEval from GATK was used to calculate Ti/Tv. In humans, the expected Ts/Tv ratio in whole-genome sequencing is around 2-2.1, within the range of our results: Ts/Tv = 2.18 for known variants and Ts/Tv = 1.91 for novel variants (Supplementary Table 3).

Supplementary Note 3. Population analysis

Relatedness

Kinship analysis was performed using KING[13] with only bi-allelic autosomal SNPs. We calculated the kinship score within the individuals of each population and between the individuals belonging to different populations. We did not find any individual related to one from another population, but we detected several 2nd and 3rd degree relations within populations (Supplementary Figure 5). We removed one individual from each of the 2nd and 3rd degree relations until we were left with only unrelated individuals (Supplementary Table 1).

Principal component analysis

We performed a Principal Component Analysis (PCA) using 50 Roma and 49 non-Roma Romanians to detect possible mislabeled individuals or some unforeseen bias (Supplementary Table 1). We converted the VCF file to PED and MAP formats using PLINK 1.9[14] keeping only bi-allelic autosomal SNPs, filtering by Minor Allele Frequency (MAF) (--maf 0.05), without missing information (--geno 0) and under Hardy-Weinberg Equilibrium (--hwe 0.000001 midp). The resulting dataset (5,216,078) was pruned (--indep 50 5 2). PCA was performed with Eigensoft (version 6.1)[15] in the remaining 515,723 SNPs (Supplementary Figure 6). The first principal component (PC1) separates a tight cluster formed by most of the non-Roma Romanian individuals from a more spread cluster of Roma individuals. We see several admixed individuals that do not clearly belong to one cluster or another; and a sample labeled as Roma clustered with non-Roma Romanians (Supplementary Figure 6). Mislabeled samples and samples that could not be clearly assigned to a cluster were removed from the main analysis (Supplementary Table 1). After removing samples indicated in Supplementary Table 1, we were left with 40 Roma and 40 non-Roma Romanians. We merged these 80 samples with worldwide populations from 1000 Genomes Project Phase 3[16]: CEU (Utah Residents (CEPH) with Northern and Western European Ancestry), TSI (Toscani in Italia), FIN (Finnish in Finland), GBR (British in England and Scotland), IBS (Iberian Population in Spain), CHB (Han Chinese in Beijing, China), JPT (Japanese in Tokyo, Japan), CHS (Southern Han Chinese), CDX (Chinese Dai in Xishuangbanna, China), KHV (Kinh in Ho Chi Minh City, Vietnam), GIH (Gujarati Indian from Houston,

Texas), PJL (Punjabi from Lahore, Pakistan), BEB (Bengali from Bangladesh), STU (Sri Lankan Tamil from the UK), ITU (Indian Telugu from the UK), YRI (Yoruba in Ibadan, Nigeria), LWK (Luhya in Webuye, Kenya), GWD (Gambian in Western Divisions in the Gambia), MSL (Mende in Sierra Leone), and ESN (Esan in Nigeria). From each population we randomly selected 40 unrelated individuals. We also added the following populations from continental India: 10 Uttar Pradesh Upper Caste Brahmins (UBR), 10 Rajput (RAJ), 10 Irula (ILA), 10 Riang (RIA), nine Birhor (BIR), and nine Vellalar (VLR) (Mondal et al. 2016). We applied the same filters as before resulting in a dataset of 938 individuals and 4,574,497 SNPs. We performed a PCA on the pruned dataset without the African populations (YRI, LWK, GWD, MSL, and ESN) (738 individuals and 467,592 SNPs). Roma are differentiated from the rest in PC3 whereas PC4 is created by two tribal Indian populations: BIR and ILA (Supplementary Figure 7).

Admixture analysis

To infer the ancestral populations of the Roma individuals, we run ADMIXTURE (version 1.3.0)[17] in the pruned dataset of 738 worldwide individuals. We tested values of K from 2 to 9 with 5-fold cross-validation (Supplementary Figure 8a). Each K was run 25 times with different seeds and the K with the lowest CV was selected. The best supported model was K = 4 (Supplementary Figure 8b). As we were not including African populations, the first split is between Asian and European components (K = 2). In K = 3 appears an Indian component that is also seen in the Roma. Indian populations show both European and Asian components along with the Indian genetic component. The Roma populations show their own component in K = 4 (main text). In K = 5 we see the distinction between the Japanese (JPT) and the Han Chinese (CHB and CHS) from other Asian populations. Finnish (FIN) show their own component in K = 6. In K = 7 the Indian component is separated in tribal (ILA and BIR) and non-tribal. In K = 8, JPT separates from the other Asian populations. In K = 9, VLR and BIR show their own component.

Supplementary Note 4. Cytokine stimulation assays

PBMC collection and stimulation experiments

After obtaining informed consent, venous blood was drawn from the cubital vein of volunteers into 10 mL EDTA tubes (Monoject) from 8 healthy donors of Caucasian (European) descent. The peripheral blood mononuclear cell (PBMC) fraction was obtained by density centrifugation of blood diluted 1:1 in pyrogen-free saline over Ficoll-Paque (Pharmacia Biotech). Cells were washed twice in saline and suspended in medium (RPMI 1640) supplemented with gentamicin 10 mg/mL, l-glutamine 10 mM and pyruvate 10 mM. Addition of antibiotics such as gentamycin is a standard methodology used to avoid contamination of cultures, and it does not influence the ability to induce cytokine production by PBMCs or macrophages (data not shown). The cells were counted in a Coulter counter (Coulter Electronics) and the number was adjusted to 5×10^{6} cells/mL. Then 5×10^{5} PBMCs in a 100µL volume were added to round-bottom 96-wells plates (Greiner) and incubated with 100 µL of stimulus. After 24h the supernatants were collected and stored at −20°C until assayed. The stimulation time periods were chosen based on extensive previous studies that showed that 24h stimulation was best suited to assess monocyte-derived cytokines [18,19]. Cytokine concentrations were measured in the supernatants by commercial ELISA (R&D Systems, Minneapolis, USA).

Supplementary Figures

Supplementary Figure 1. Distribution of the average coverage for autosomal chromosomes in a) Roma and b) non-Roma Romanian samples. Fraction of the genome that is covered by at least X reads in c) Roma and d) Romanian samples.

a)

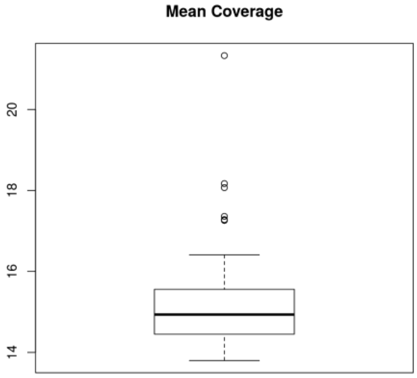

b)

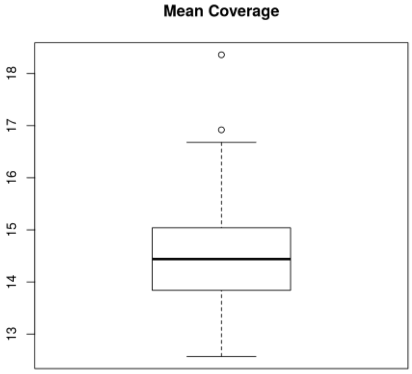

c)

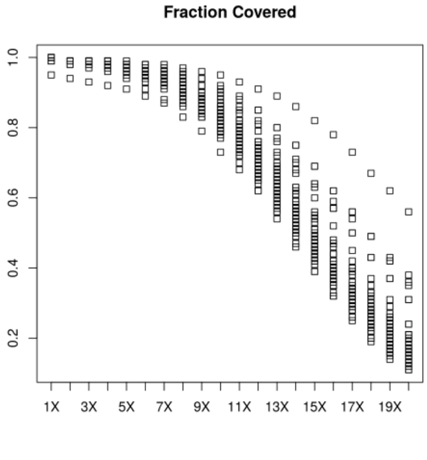

d)

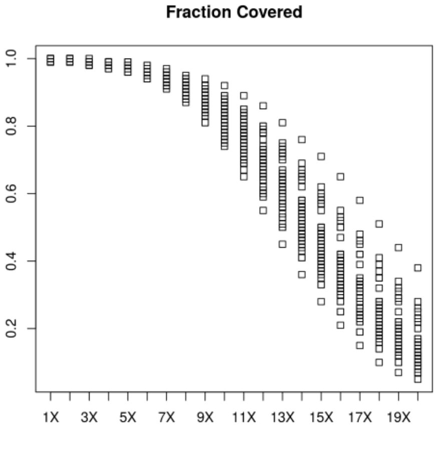

Supplementary Figure 2. Ratio of the coverage on the X and Y chromosomes with respect to the coverage on autosomal chromosomes for Roma and non-Roma Romanian samples. Sample RMN-17 with ambiguous sex determination is labelled. For Romanian samples are classified as female: RMN-7, RMN-12, RMN-14, and RMN-31.

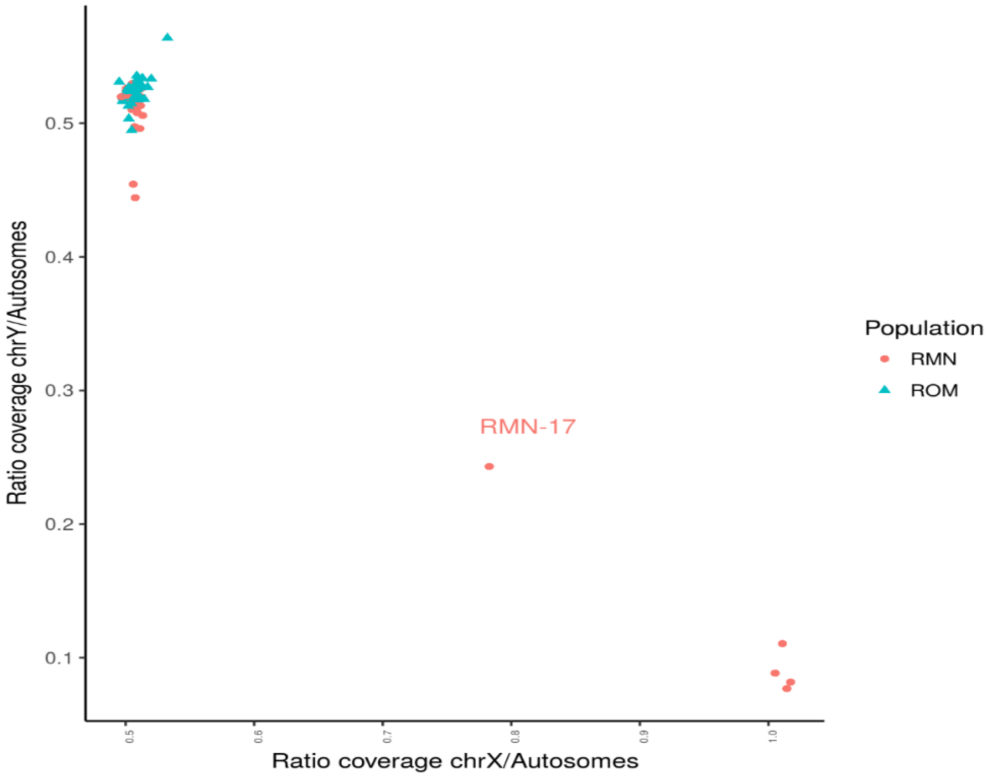

Supplementary Figure 3. a) Inbreeding coefficient (F) for Roma (ROM) and non-Roma Romanian (RMN) samples. b) Same plot as a) after removing outlier sample RMN-17.

a)

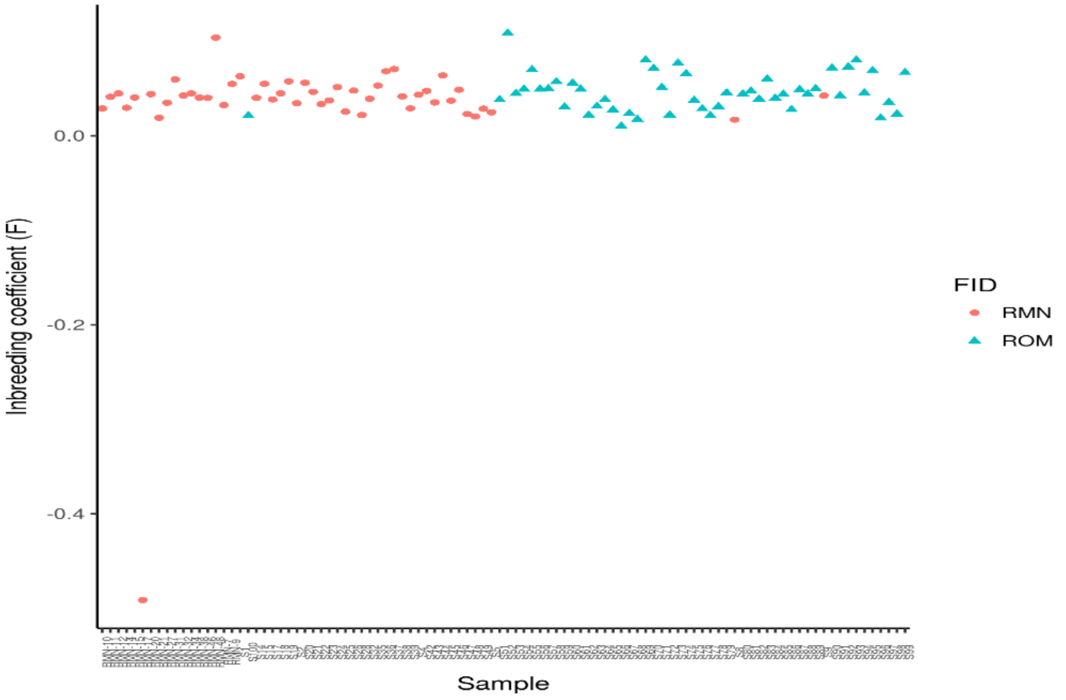

b)

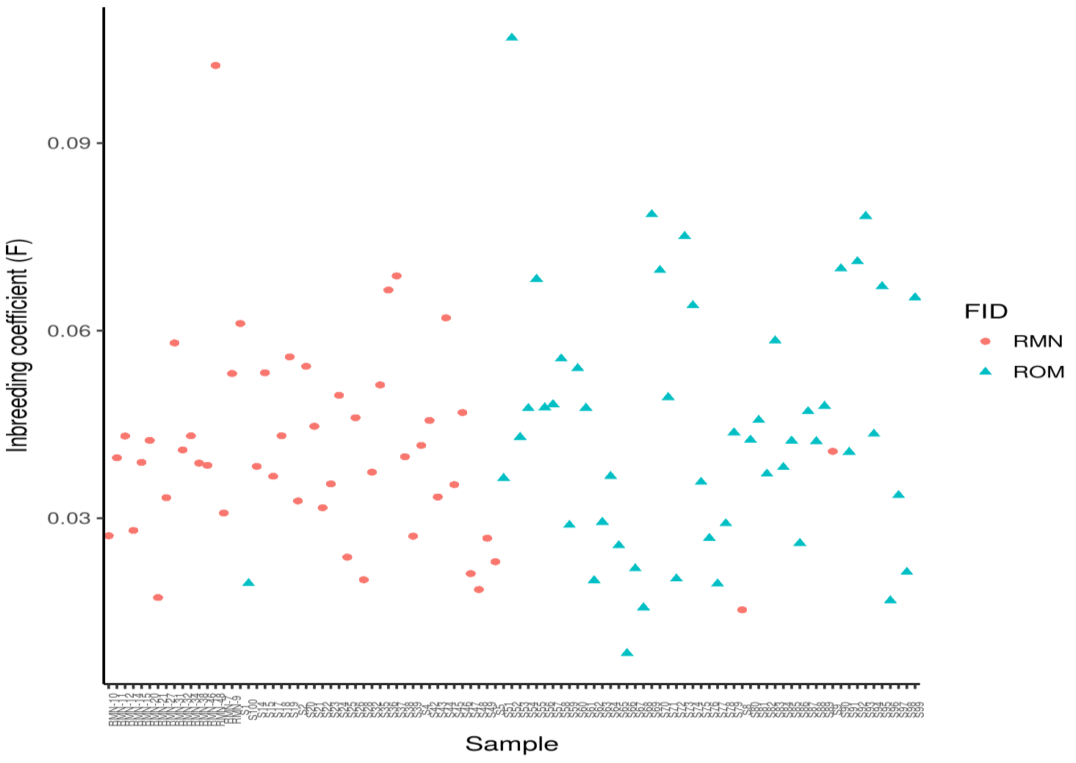

Supplementary Figure 4. Estimates of mtDNA contamination in Roma and non-Roma Romanian samples. Samples with an estimated percentage of contaminant genome over 5% were classified as contaminated.

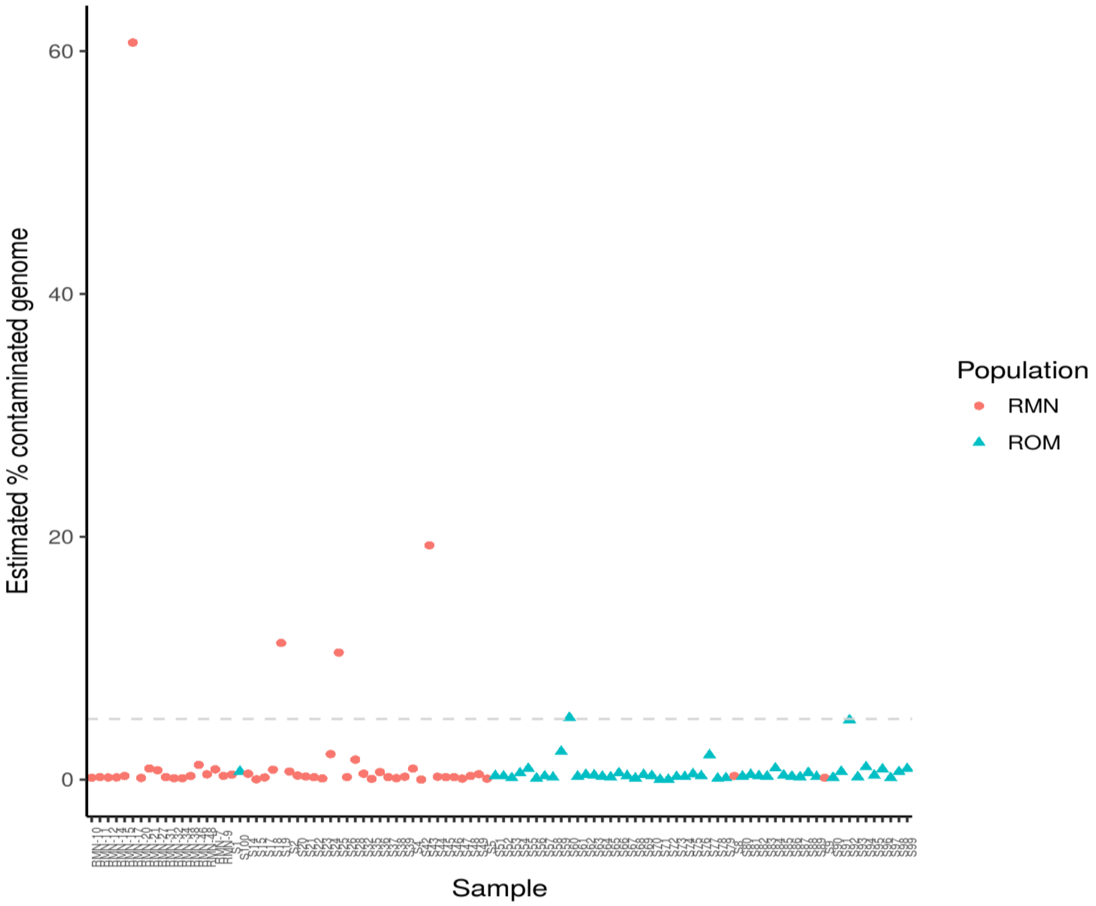

Supplementary Figure 5. Estimated kinship coefficient versus the proportion of SNPs with zero Identical-by-state (IBS0) in 50 Roma (ROM) and 49 non-Roma Romanian (RMN; without contaminated sample RMN-17). Blue dashed line indicates threshold of 2nd degree relation (Kinship range = [0.0884, 0.177]); grey dashed line indicates threshold of 3rd degree relation (Kinship range = [0.0442, 0.0884]).

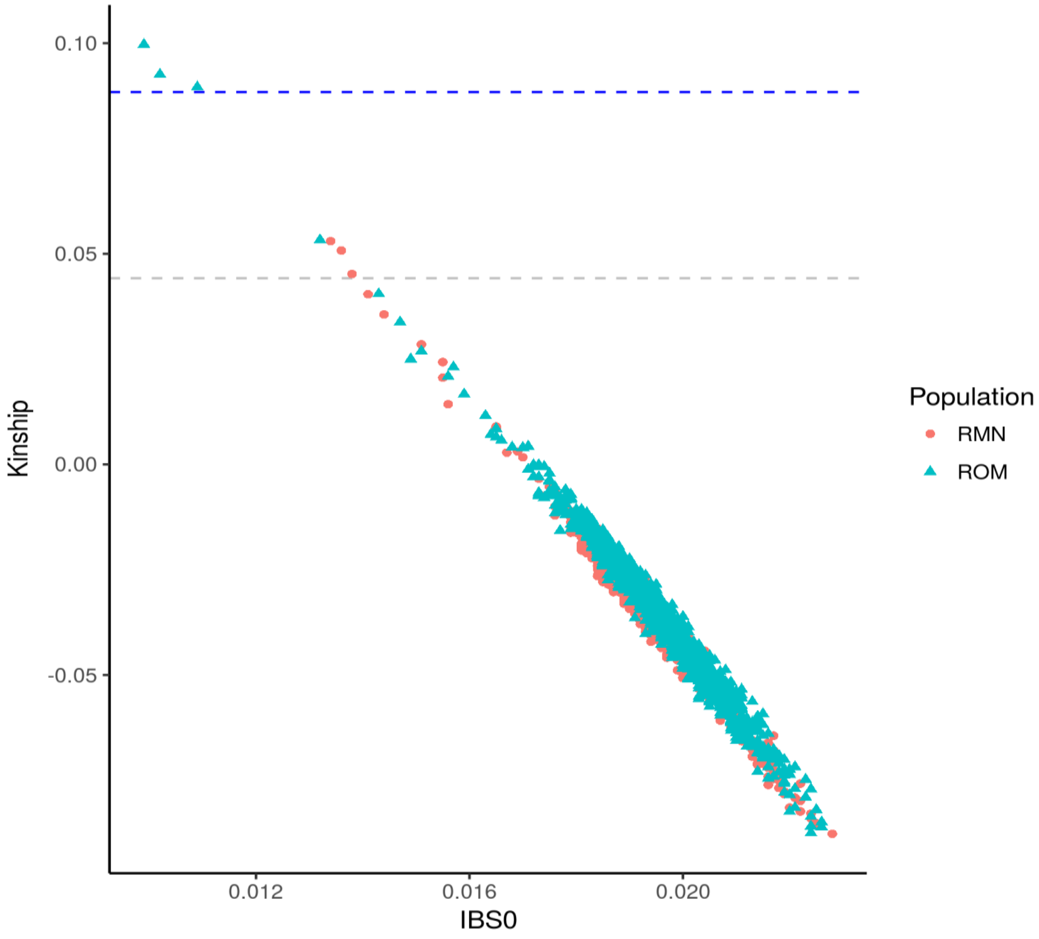

Supplementary Figure 6. Principal component analysis of 50 Roma (ROM) and 50 non-Roma Romanian samples (RMN). Principal component (PC) 1 and PC2. The percentage of variance explained by each component is added in the labels.

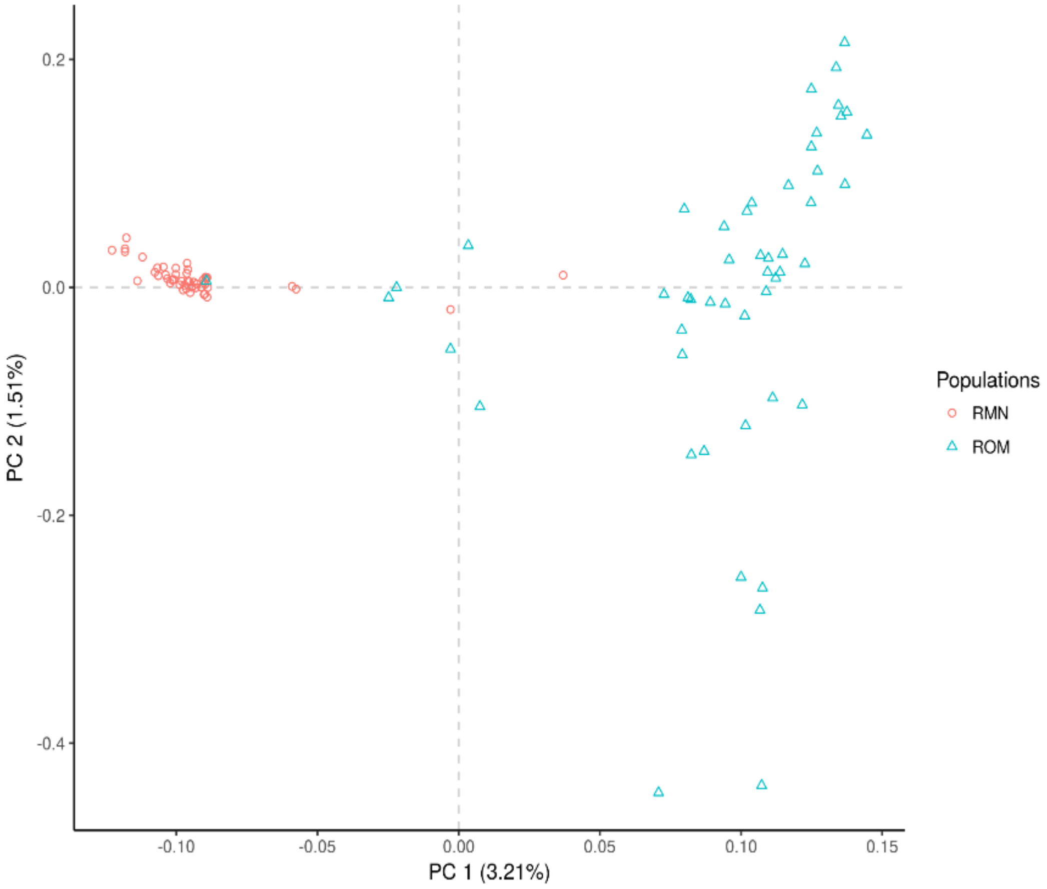

Supplementary Figure 7. Principal component analysis of 40 Roma (ROM) and 40 non-Roma Romanian (RMN) samples with 1000 Genomes Project Phase 3 and mainland Indian populations. Principal component (PC) 1 and PC2 are shown in the main text. PC 3 and PC4. The percentage of variance explained by each component is added in the labels.

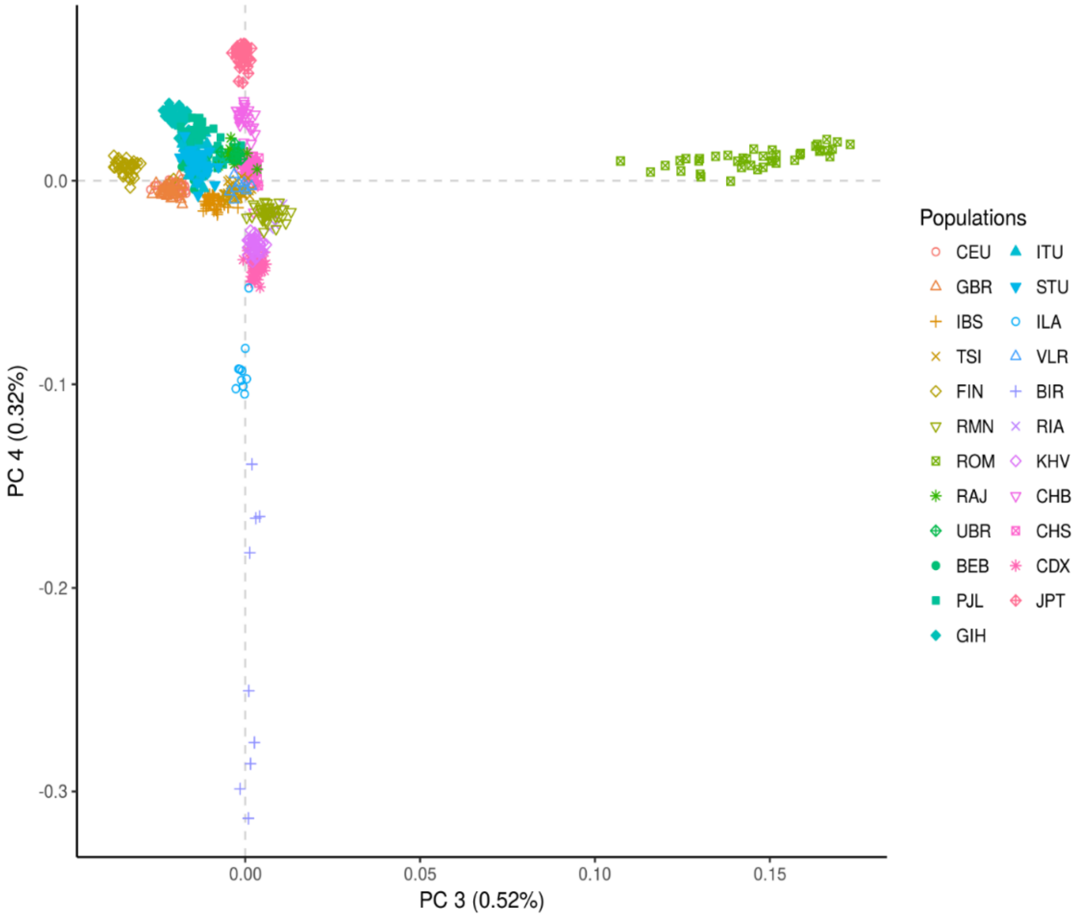

Supplementary Figure 8. Admixture plot of 40 Roma (ROM) and 40 non-Roma Romanian (RMN) with 1000 Genomes Project Phase 3 and mainland Indian populations. a) Admixture plot showing runs of K from 2 to 9; b) Cross-validation error of runs with K values from 2 to 9.

a)

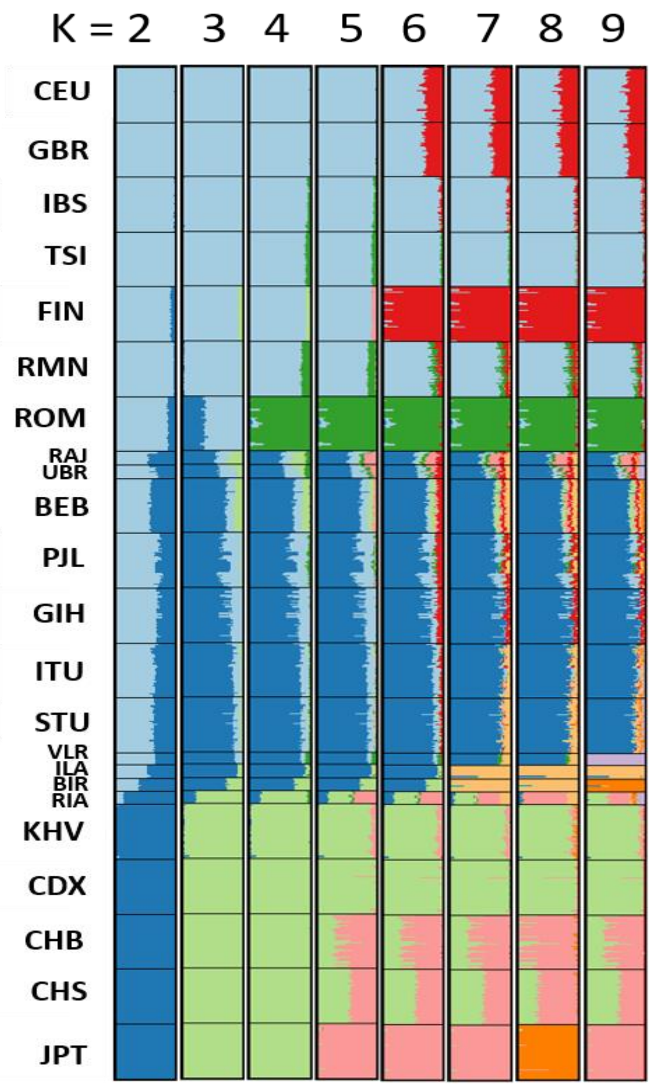

b)

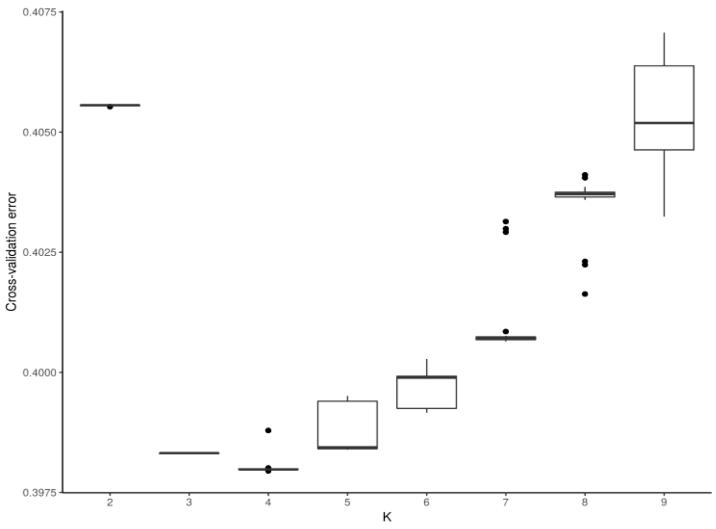

Supplementary Figure 9. Outgroup f3-statistics f3(Roma; X, YRI), without merging with 1000 Genomes data.

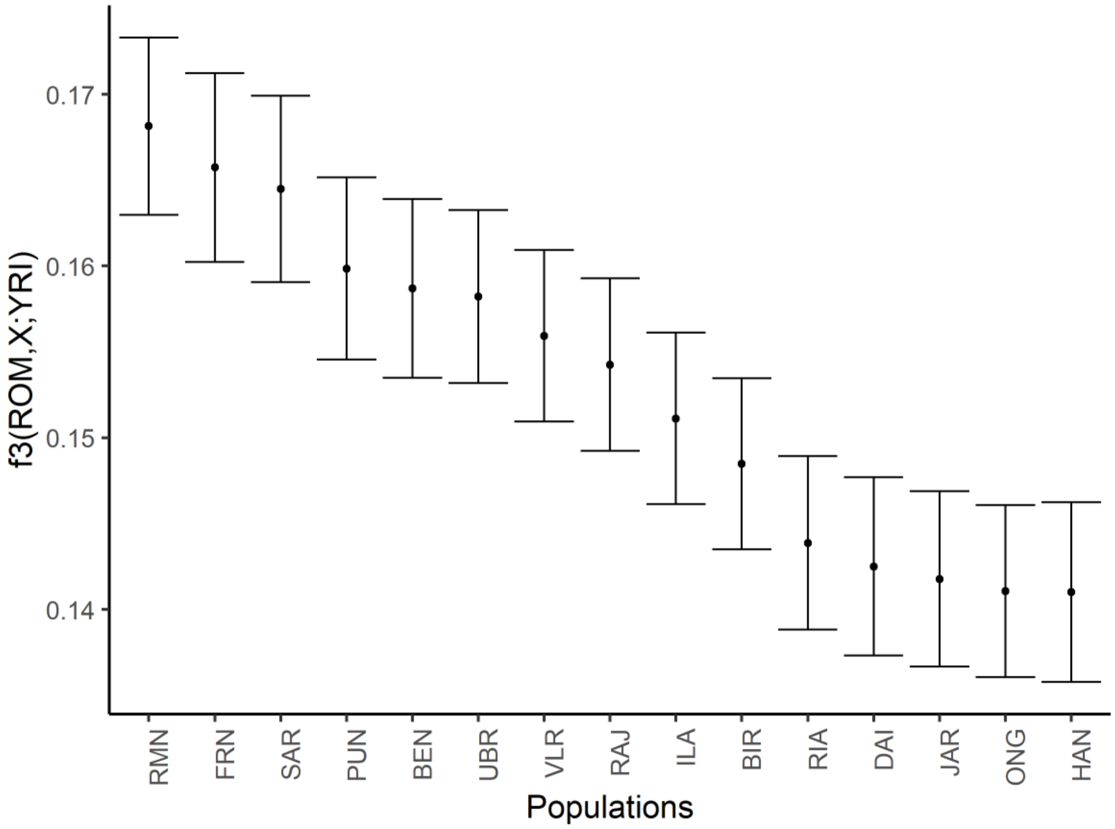

Supplementary Figure 10. Runs of homozygosity in worldwide populations. a) Mean total length of ROH (Mb) in a population according to a given ROH length. ROH were classified in four categories: 1 ≤ ROH < 2 Mb, 2 ≤ ROH < 4 Mb, 4 ≤ ROH < 8 Mb and ROH ≥ 8 Mb. Roma population is highlighted in black; b) Same comparison but all populations were randomly sampled to match the lowest sample size available (n = 9); c) Total number of runs of homozygosity (NROH) versus the sum of the total length of ROH in Mb (SROH) for ROH > 2 Mb in worldwide populations. Each small dot represents an individual, while big dots represent population means. Except for Roma who are colored in orange, dots color denotes their geography: Europe (pink), South Asia (blue) and East Asia (green). Consanguinity increases the variance in SROH, as seen by the shift to the right of several individuals in ROM, ILA, BIR, RIA and VLR populations; d) Total number of runs of homozygosity (NROH) versus the sum of the total length of ROH in Mb (SROH) for ROH > 2 Mb in worldwide populations. Each dot represents population means. Except for Roma who are colored in orange, dots color denotes their geography: Europe (pink), South Asia (blue) and East Asia (green). Right panel is an inset of the main plot to better show the separation between populations. All populations were randomly sampled to match the lowest sample size available (n = 9). See Figure 2 for the comparison with populations with different sample sizes. See Supplementary Notes for the description of the population abbreviations.

a)

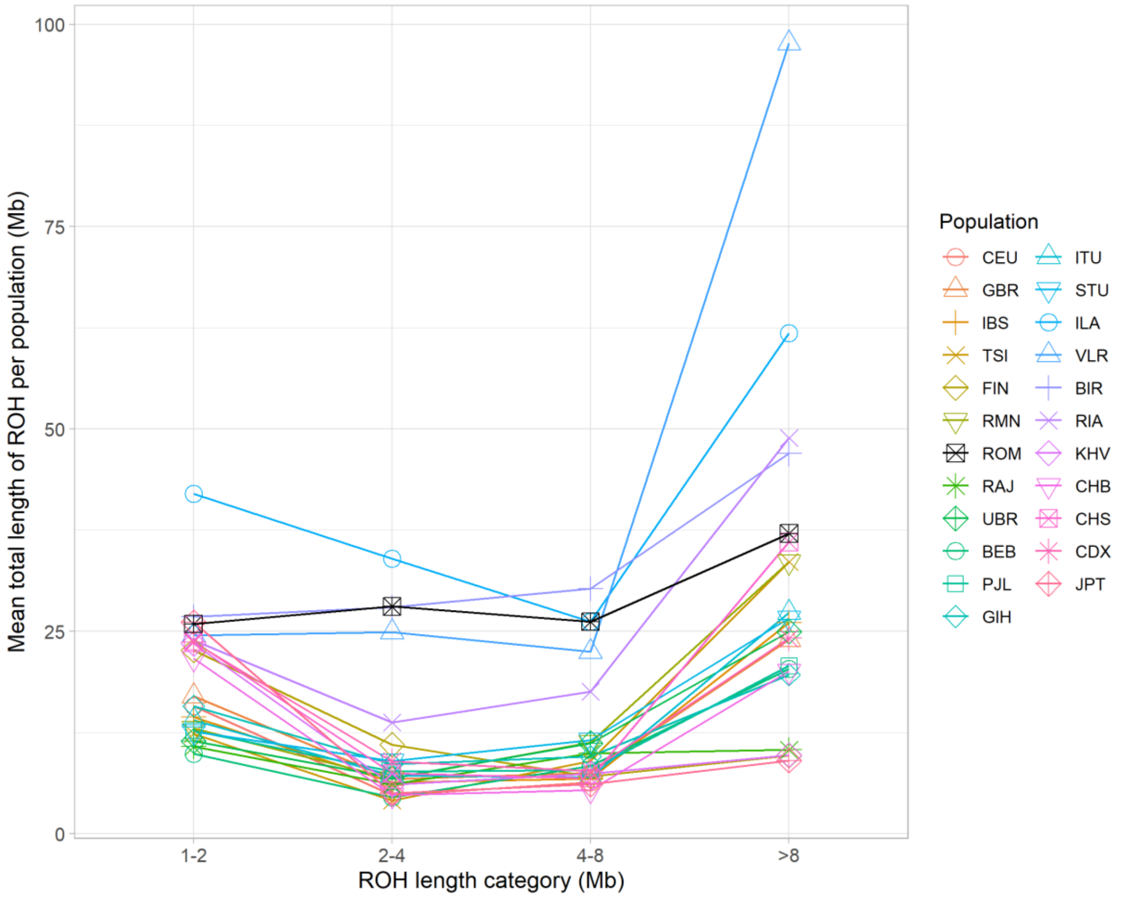

b)

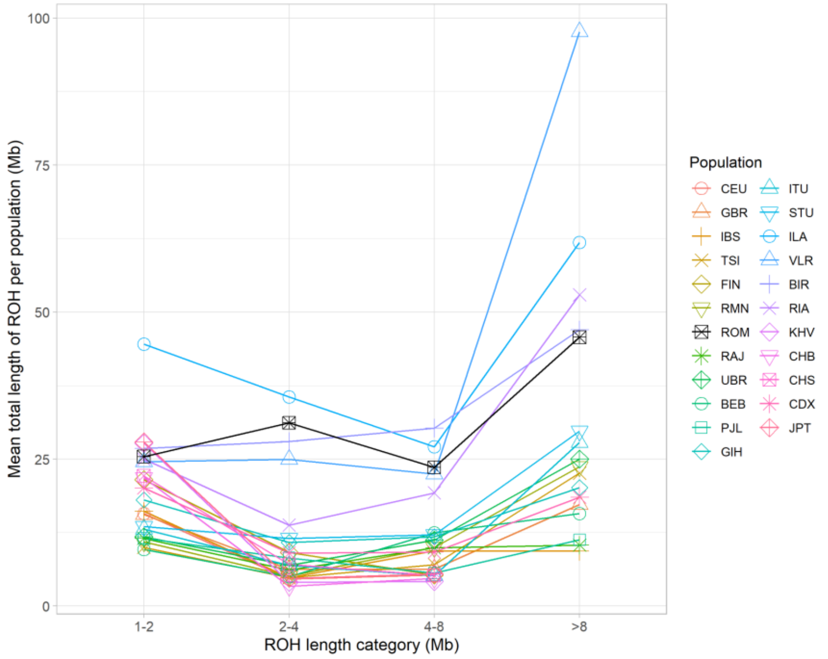

Mean total length of ROH per population (Mb)
100
75
50
25
0
1-2
2-4
4-8
>8
ROH length category (Mb)
Population
CEU
GBR
IBS
TSI
FIN
RMN
ROM
RAJ
UBR
BEB
PJL
GIH
ITU
STU
ILA
VLR
BIR
RIA
KHV
CHB
CHS
CDX
JPT

c)

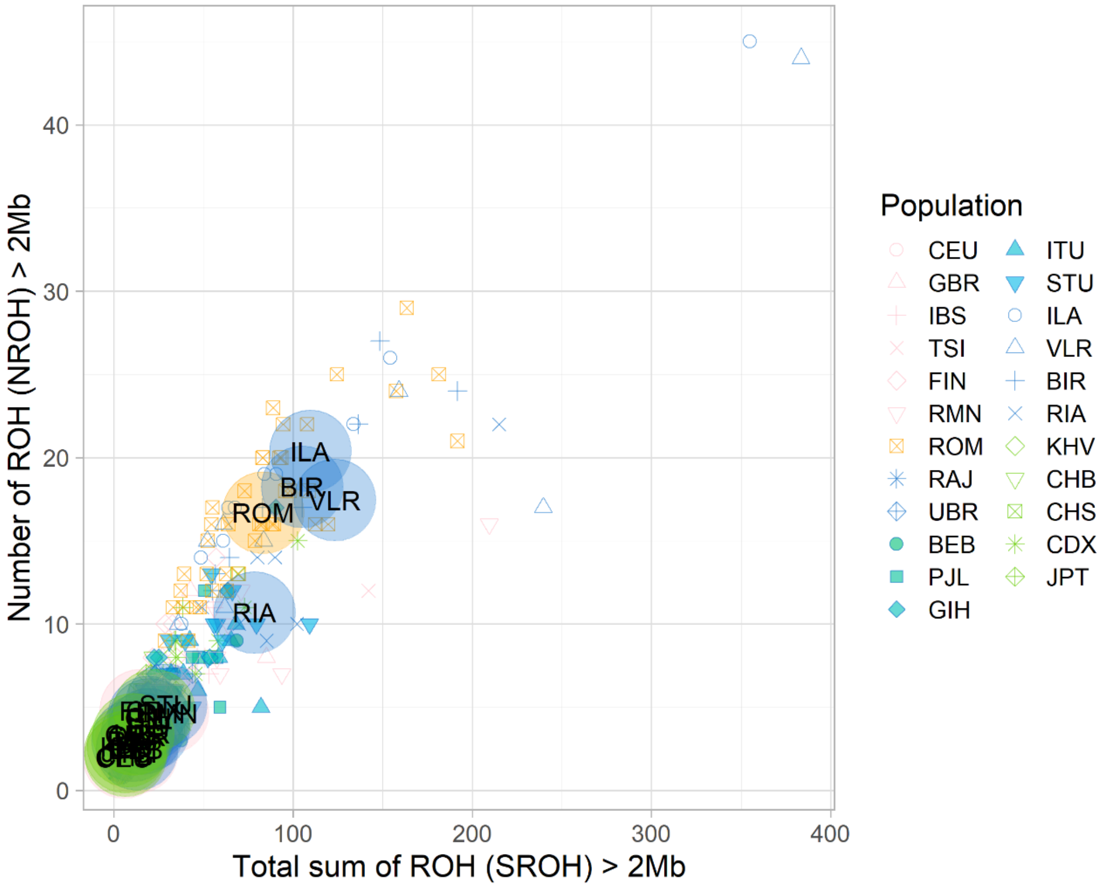

Number of ROH (NROH) > 2Mb
40
30
20
10
0
0
100
200
300
400
Total sum of ROH (SROH) > 2Mb
ILA
BIR
VLR
ROM
RIA
Population
CEU
GBR
IBS
TSI
FIN
RMN
ROM
RAJ
UBR
BEB
PJL
GIH
ITU
STU
ILA
VLR
BIR
RIA
KHV
CHB
CHS
CDX
JPT

d)

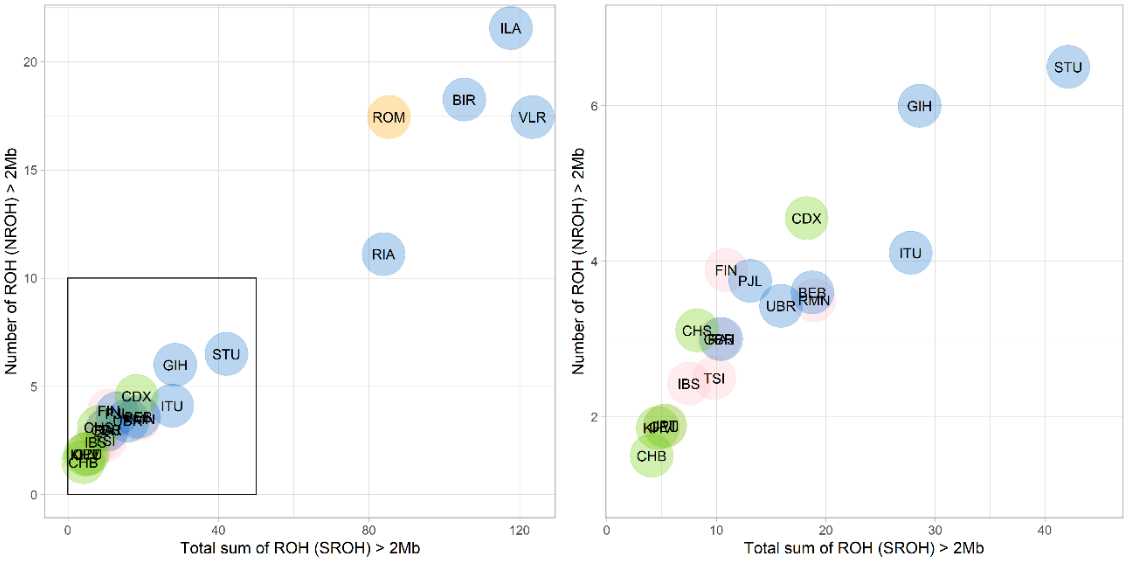

Number of ROH (NROH) > 2Mb
Total sum of ROH (SROH) > 2Mb
ILA
BIR
ROM
VLR
RIA
STU
GIH
CDX
ITU
FIN
PJL
UBR
CHS
IBS
TSI
CHB
Number of ROH (NROH) > 2Mb
Total sum of ROH (SROH) > 2Mb

Supplementary Figure 11. Effective population size change through time in Roma and non-Roma Romanians. Estimated effective population size (Ne) is represented by a dashed line and the shade is the 95% confidence interval. Generation time is assumed to be 25 years.

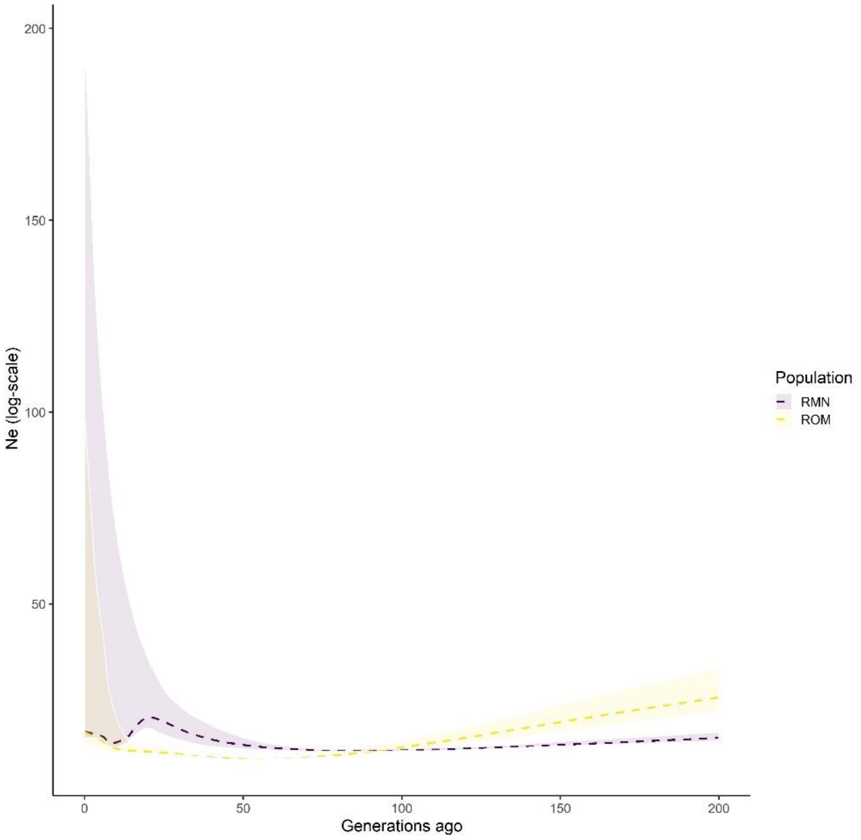

Supplementary Figure 12. Pathway enrichment analysis based on the genes under selection. Extension of main Figure 3b. Bar length is proportional to the percentage of genes in the term found within our signals, while the number of genes found in each term is shown. All terms are statistically significant (p-value < 0.05, Benjamini and Hochberg - False Discovery Rate (FDR). Terms with an excess of European local ancestry are marked with an asterisk next to the name (p-value < 0.05, Benjamini and Hochberg - FDR).

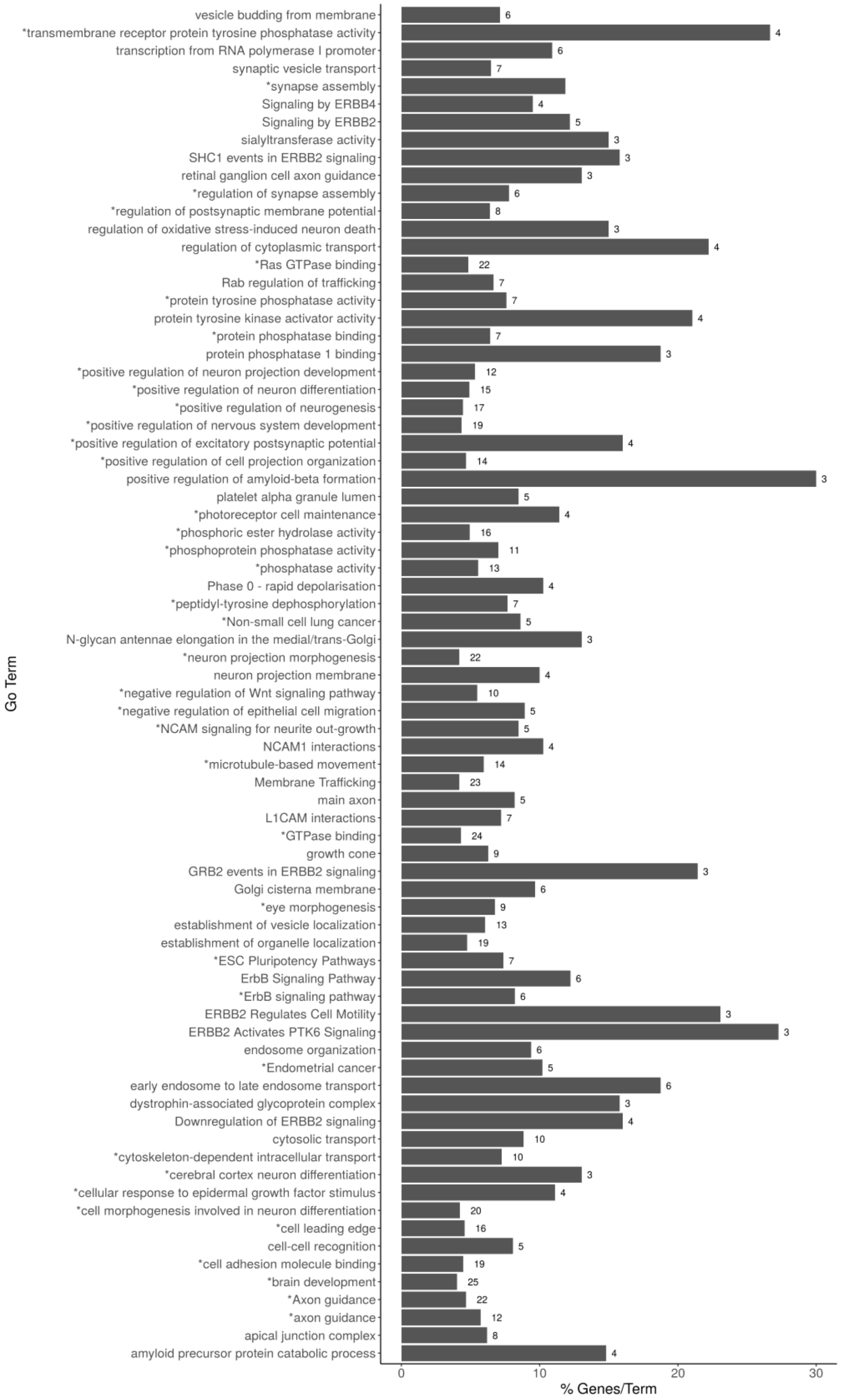

Supplementary Figure 13. Number of cQTLs found in the selection Signals (blue line) compared to permutation results (grey histograms). Extension of main Figure 4B with different thresholds for the cQTL analysis (top to bottom: p<=1e-10, 1e-8, 1e-7, 1e-6, 1e-5, 1e-4) and two percentages for the selection signals (2% and 5%). The overlap was compared to 10,000 random permutations.

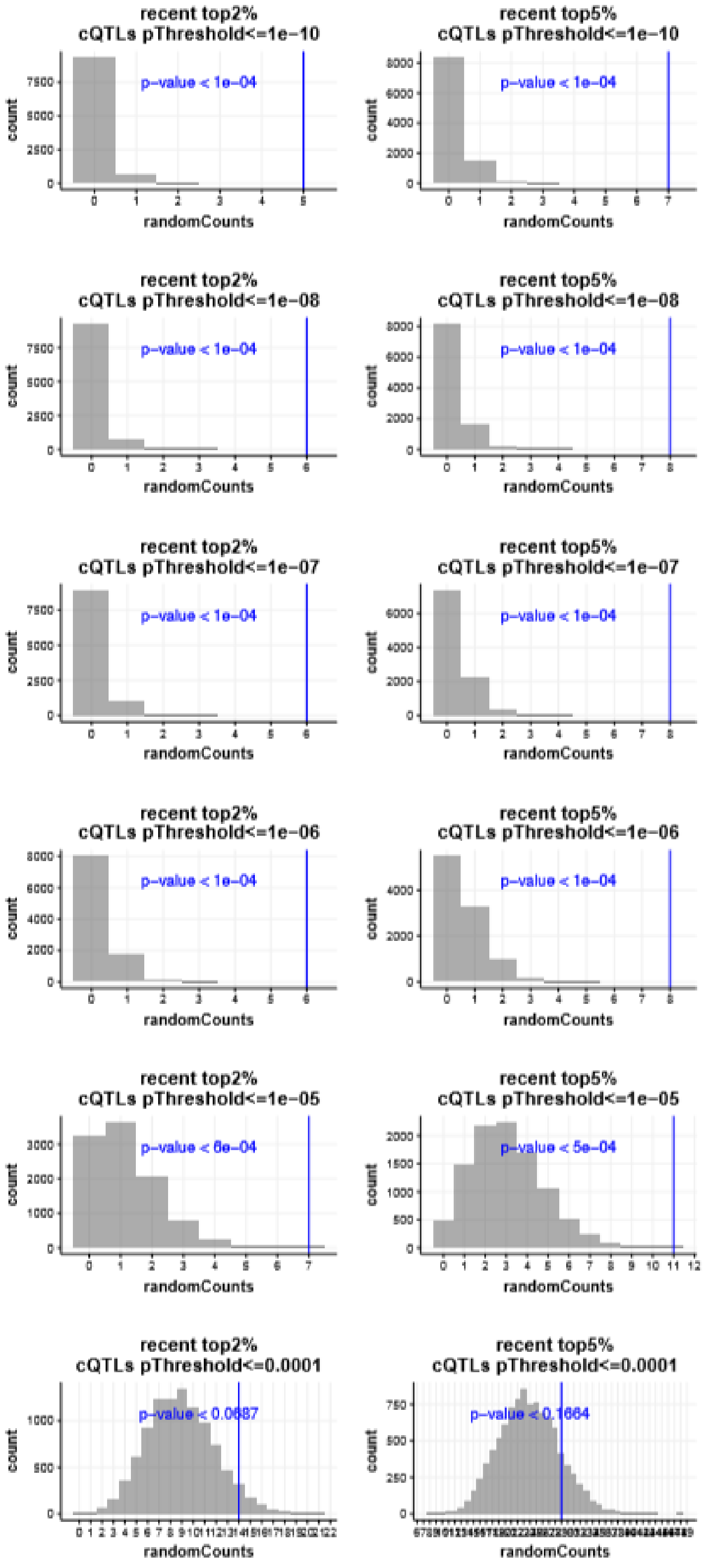

Supplementary Tables

Supplementary Table 1. Romanian samples removed from the main analysis and reason for exclusion.

Sample	Population	Reason
RMN-17	Non-Roma	mtDNA contamination; outlier X heterozygosity; outlier autosomal heterozygosity
S19	Non-Roma	mtDNA contamination
S25	Non-Roma	mtDNA contamination
S43	Non-Roma	mtDNA contamination
S60	Roma	mtDNA contamination
S92	Roma	2nd-degree relation to S9 (Roma)
S85	Roma	2nd-degree relation to S87 (Roma)
S71	Roma	2nd-degree relation to S72 (Roma); Admix individual
S72	Roma	2nd-degree relation to S71; Admix individual
S79	Roma	Discordant self-identification
S74	Roma	3rd-degree relation to S70 (Roma)
S21	Roma	3rd-degree relation to S9 and S17 (Roma)
S8	Roma	3rd-degree relation to S14 (Roma)
S66	Roma	Admix individual
S96	Roma	Admix individual
S100	Roma	Admix individual
RMN-11	Non-Roma	Admix individual
RMN-14	Non-Roma	Admix individual
S28	Non-Roma	Admix individual
RMN-21	Non-Roma	Admix individual

Supplementary Table 2. Estimation of X-chromosome heterozygosity in male samples due to contamination. ML = Maximum likelihood contamination estimate; SE = standard error estimated using jackknife.

**Sample**	**Putative source of contamination**	**ML**	**SE(ML)**
RMN-17	CEU	0.438325	0.003455738
RMN-17	CHB	0.359364	0.00285054
RMN-17	GIH	0.392264	0.003129929
RMN-17	PEL	0.343802	0.002722593
RMN-17	YRI	0.335109	0.00266473

Supplementary Table 3. Transitions versus transversions ratio for Roma and non-Roma Romanian individuals. Reported values for all, known and novel variants. Novel variants are defined by using dbSNP137.

**Marker**	**nTs**	**nTv**	**Ts/Tv**
All	14516088	6988721	2.08
Known	9552023	4382784	2.18
Novel	4964065	2605937	1.91

Supplementary Table 4. Best predicted mitochondrial haplotypes per individual.

Sample	Predicted Haplogroup	Population
S1	A12a	Non-Roma Romanian
S14	H11a2	Non-Roma Romanian
S42	H1b1b	Non-Roma Romanian
RMN-31	H1g2	Non-Roma Romanian
S37	H21	Non-Roma Romanian
S36	H44a	Non-Roma Romanian
S45	H55	Non-Roma Romanian
S46	H55	Non-Roma Romanian
RMN-34	H5e1	Non-Roma Romanian
RMN-48	H5e1a1	Non-Roma Romanian
S44	H7	Non-Roma Romanian
RMN-20	H79	Non-Roma Romanian
RMN-46	HV0	Non-Roma Romanian
RMN-15	J1c_16261	Non-Roma Romanian
RMN-32	J1c_16261	Non-Roma Romanian
RMN-10	J1c3k	Non-Roma Romanian
S26	K1b1c	Non-Roma Romanian
S35	K1c1a	Non-Roma Romanian
S38	K1c1a	Non-Roma Romanian
RMN-7	L0a1a1	Non-Roma Romanian
S47	M7b1a1a1	Non-Roma Romanian
S23	T1a1l	Non-Roma Romanian
S39	T1a1l	Non-Roma Romanian
S5	T2a1a1	Non-Roma Romanian
S18	T2b	Non-Roma Romanian
S22	T2b	Non-Roma Romanian
S49	T2b	Non-Roma Romanian
S20	T2f2	Non-Roma Romanian
S48	U3b	Non-Roma Romanian
RMN-27	U4c1a	Non-Roma Romanian
RMN-12	U5a1a1	Non-Roma Romanian
S32	U5a1b	Non-Roma Romanian
S17	U5a1c2a	Non-Roma Romanian
S9	U5a1c2a	Non-Roma Romanian
S24	U5a2a1b	Non-Roma Romanian
S2	U5a2b1c	Non-Roma Romanian

S4	U5b1_16192	Non-Roma Romanian
RMN-38	W1_119;W7	Non-Roma Romanian
RMN-9	W3b	Non-Roma Romanian
S15	X2e1b	Non-Roma Romanian
S75	H;H3_16189;H1_16189	Romanian Roma
S88	H;H3_16189;H1_16189	Romanian Roma
S89	H;H3_16189;H1_16189	Romanian Roma
S95	H;H3_16189;H1_16189	Romanian Roma
S98	H;H3_16189;H1_16189	Romanian Roma
S54	H11a2	Romanian Roma
S51	H20	Romanian Roma
S62	H40b	Romanian Roma
S86	H44b	Romanian Roma
S65	H5a1a	Romanian Roma
S53	H5a2	Romanian Roma
S87	H5a2	Romanian Roma
S99	H5b4	Romanian Roma
S52	H7a1a	Romanian Roma
S94	H7a1a	Romanian Roma
S68	I1a1a	Romanian Roma
S90	I1a1a	Romanian Roma
S58	J1b3a	Romanian Roma
S57	J1c2e	Romanian Roma
S63	K2a6	Romanian Roma
S55	M35b2	Romanian Roma
S67	M35b2	Romanian Roma
S56	M5a1b	Romanian Roma
S59	M5a1b	Romanian Roma
S61	M5a1b	Romanian Roma
S64	M5a1b	Romanian Roma
S69	M5a1b	Romanian Roma
S73	M5a1b	Romanian Roma
S80	M5a1b	Romanian Roma
S81	M5a1b	Romanian Roma
S82	M5a1b	Romanian Roma
S83	M5a1b	Romanian Roma
S84	M5a1b	Romanian Roma
S91	M5a1b	Romanian Roma
S93	M5a1b	Romanian Roma

S97	M5a1b	Romanian Roma
S77	U3a1a	Romanian Roma
S76	W3b	Romanian Roma
S78	X2d1	Romanian Roma
S70	X2e1;X2e;X2e1b;X2m'n	Romanian Roma

Supplementary Table 5. European admixture in the Roma. D-statistic test in the form of D(European, African(YRI), ROM, South Asian).

**W (European)**	**X (African)**	**Y (Roma)**	**Z (South Asian)**	**D-statistic**	**Standard error**	**Z-score**
RMN	YRI	ROM	RAJ	0.0341	0.00170	20.0
FRN	YRI	ROM	RAJ	0.0308	0.00222	13.9
SAR	YRI	ROM	RAJ	0.0348	0.00224	15.5
RMN	YRI	ROM	UBR	0.0239	0.00156	15.4
FRN	YRI	ROM	UBR	0.0245	0.00224	11.0
SAR	YRI	ROM	UBR	0.0282	0.00210	13.5
RMN	YRI	ROM	ILA	0.0533	0.00195	27.4
FRN	YRI	ROM	ILA	0.0537	0.00286	18.8
SAR	YRI	ROM	ILA	0.053	0.00279	19.0
RMN	YRI	ROM	VLR	0.0367	0.00182	20.2
FRN	YRI	ROM	VLR	0.0373	0.00256	14.6
SAR	YRI	ROM	VLR	0.0403	0.00258	15.6
RMN	YRI	ROM	BIR	0.0618	0.00212	29.1
FRN	YRI	ROM	BIR	0.0609	0.00292	20.9
SAR	YRI	ROM	BIR	0.0648	0.00304	21.3

Supplementary Table 6. Proportion of West European ancestry in the Roma. Estimated by the f4 ratio estimation where α = f4(YRI,EUR,ROM,RAJ) / f4(YRI,EUR,RMN,RAJ).

**A**	**B**	**X**	**C**	**O**	**alpha**	**Standard error**	**Z-score**
YRI	RMN	ROM	RAJ	CEU	0.482	0.0140	34.3
YRI	RMN	ROM	RAJ	GBR	0.485	0.0141	34.3
YRI	RMN	ROM	RAJ	IBS	0.526	0.0131	40.2
YRI	RMN	ROM	RAJ	TSI	0.538	0.0138	38.9

Supplementary Table 7. Candidate genes and nonsynonymous (nonsyn) candidate variants in Recent Shared Signals. Locations are in GRCh37 (hg19).

**Marker**	**Chr**	**Position**	**Ancestral**	**Derived**	**Gene**	**Type**	**% European ancestry**
rs614486	chr1	47138819	T	G	*TEX38*	nonsyn	71.43
rs2056899	chr1	47607851	A	T	*CYP4A22*	nonsyn	71.43
rs1056820	chr13	41515286	T	A	*ELF1*	nonsyn	58.44
rs7799	chr13	41533052	T	C	*ELF1*	nonsyn	58.44
rs2287679	chr19	33600764	T	C	*GPATCH1*	nonsyn	84.42
rs10416265	chr19	33605300	A	G	*GPATCH1*	nonsyn	84.42
rs10421769	chr19	33605312	T	C	*GPATCH1*	nonsyn	84.42
rs1402467	chr2	108994808	C	G	*SULT1C4*	nonsyn	81.81
rs59900519	chr2	135988127	T	A	*ZRANB3*	nonsyn	76.62
rs935615	chr2	135988416	C	T	*ZRANB3*	nonsyn	76.62
rs1112438	chr3	39152345	G	A	*TTC21A*	nonsyn	67.53
rs1453241	chr3	130103709	G	A	*COL6A5*	nonsyn	83.12
rs11917356	chr3	130110550	A	G	*COL6A5*	nonsyn	83.12
rs12488457	chr3	130116696	A	C	*COL6A5*	nonsyn	83.12
rs1497312	chr3	130125116	G	C	*COL6A5*	nonsyn	83.12
rs16827497	chr3	130134492	T	C	*COL6A5*	nonsyn	83.12
rs3762672	chr3	132218623	G	T	*DNAJC13*	nonsyn	66.23
rs34358	chr5	74965122	G	A	*ANKDD1B*	stopgain	85.71
rs2307111	chr5	75003678	T	C	*POC5*	nonsyn	85.71
rs1550526	chr6	13295515	A	C	*LOC100130357*	nonsyn	83.12
rs2305473	chr7	158536267	T	C	*ESYT2*	nonsyn	94.81
rs2305475	chr7	158536345	A	G	*ESYT2*	nonsyn	94.81
rs2788478	chr7	158672619	A	G	*WDR60*	nonsyn	93.51
rs7019716	chr9	26116150	G	T	*LOC100506422*	nonsyn	81.82
rs1056899	chr9	135139901	T	C	*SETX*	nonsyn	80.52

rs2296871	chr9	135173685	T	C	*SETX*	nonsyn	80.52
rs543573	chr9	135202829	T	C	*SETX*	nonsyn	80.52
rs1183768	chr9	135203231	C	T	*SETX*	nonsyn	80.52
rs1185193	chr9	135203409	A	C	*SETX*	nonsyn	80.52

Supplementary Table 8. R^2 values for the 8 SNPs in the selection signals that are also cQTLs (p-value cutoff of cQTL = 1e-7). R^2 values were calculated based on the 500FG Human Functional Genomics Project[20].

chrom-pos	4-38743114	4-38744489	4-38745482	4-38746461	4-38768669	4-38771368	4-38778903	4-38782221
4-38743114	1	0.587	0.119	0.306	0.133	0.284	0.138	0.235
4-38744489	0.587	1	0.399	0.538	0.341	0.487	0.238	0.440
4-38745482	0.119	0.399	1	0.202	0.482	0.239	0.134	0.228
4-38746461	0.306	0.538	0.202	1	0.258	0.329	0.305	0.448
4-38768669	0.133	0.341	0.482	0.258	1	0.491	0.236	0.497
4-38771368	0.284	0.487	0.239	0.329	0.491	1	0.509	0.687
4-38778903	0.138	0.238	0.134	0.305	0.236	0.509	1	0.500
4-38782221	0.235	0.440	0.228	0.448	0.497	0.687	0.500	1

References

1. Mondal, M. *et al.* Genomic analysis of Andamanese provides insights into ancient human migration into Asia and adaptation. *Nat. Genet.* **48**, 1066–1070 (2016).
2. Meyer, M. *et al.* A High-Coverage Genome Sequence from an Archaic Denisovan Individual. *Science (80-.).* **338**, 222–226 (2012).
3. Li, H. Aligning sequence reads, clone sequences and assembly contigs with BWA-MEM. *arXiv Prepr. arXiv* **00**, 3 (2013).
4. Li, H. *et al.* The Sequence Alignment/Map format and SAMtools. *Bioinformatics* **25**, 2078–2079 (2009).
5. McKenna, A. *et al.* The genome analysis toolkit: A MapReduce framework for analyzing next-generation DNA sequencing data. *Genome Res.* **20**, 1297–1303 (2010).
6. Abecasis, G. R. *et al.* An integrated map of genetic variation from 1,092 human genomes. *Nature* **491**, 56–65 (2012).
7. Sherry, S. T. dbSNP: the NCBI database of genetic variation. *Nucleic Acids Res.* **29**, 308–311 (2001).
8. Danecek, P. *et al.* The variant call format and VCFtools. *Bioinformatics* **27**, 2156–2158 (2011).
9. Korneliussen, T. S., Albrechtsen, A. & Nielsen, R. ANGSD: Analysis of Next Generation Sequencing Data. *BMC Bioinformatics* **15**, 356 (2014).
10. Fu, Q. *et al.* A revised timescale for human evolution based on ancient mitochondrial genomes. *Curr. Biol.* **23**, 553–559 (2013).
11. Johnson, P. contamMix: Mitochondrial genome contamination estimation. (2014).
12. Edgar, R. C. MUSCLE: Multiple sequence alignment with high accuracy and high throughput. *Nucleic Acids Res.* **32**, 1792–1797 (2004).
13. Manichaikul, A. *et al.* Robust relationship inference in genome-wide association studies. *Bioinformatics* **26**, 2867–2873 (2010).
14. Chang, C. C. *et al.* Second-generation PLINK: rising to the challenge of larger and richer datasets. *Gigascience* **4**, 7 (2015).

15. Patterson, N., Price, A. L. & Reich, D. Population Structure and Eigenanalysis. *PLoS Genet.* **2**, e190 (2006).
16. Gibbs, R. A. *et al.* A global reference for human genetic variation. *Nature* **526**, 68–74 (2015).
17. Alexander, D. H., Novembre, J. & Lange, K. Fast model-based estimation of ancestry in unrelated individuals. *Genome Res.* **19**, 1655–1664 (2009).
18. Netea, M. G. *et al.* A SEMI-QUANTITATIVE REVERSE TRANSCRIPTASE POLYMERASE CHAIN REACTION METHOD FOR MEASUREMENT OF MRNA FOR TNF-α AND IL-1β IN WHOLE BLOOD CULTURES: ITS APPLICATION IN TYPHOID FEVER AND EXENTRIC EXERCISE. *Cytokine* **8**, 739–744 (1996).
19. van Crevel, R. *et al.* Disease-specific ex vivo stimulation of whole blood for cytokine production: applications in the study of tuberculosis. *J. Immunol. Methods* **222**, 145–153 (1999).
20. Li, Y. *et al.* A Functional Genomics Approach to Understand Variation in Cytokine Production in Humans. *Cell* **167**, 1099-1110.e14 (2016).
